# Nintedanib inhibits growth of human prostate carcinoma cells by modulating both cell cycle and angiogenesis regulators

**DOI:** 10.1038/s41598-018-27831-1

**Published:** 2018-06-22

**Authors:** Raquel Frenedoso da Silva, Deepanshi Dhar, Komal Raina, Dileep Kumar, Rama Kant, Valeria Helena Alves Cagnon, Chapla Agarwal, Rajesh Agarwal

**Affiliations:** 1 Department of Pharmaceutical Sciences, Skaggs School of Pharmacy and Pharmaceutical Sciences, Aurora, Colorado USA; 2 University of Colorado Cancer Center, University of Colorado Anschutz Medical Campus, Aurora, Colorado USA; 3 Department of Structural and Functional Biology, Institute of Biology, University of Campinas (UNICAMP), Sao Paulo, Brazil

## Abstract

Prostate cancer (PCa) is the most common malignancy and second leading cause of cancer-related deaths in American men. Proliferating cells have higher need for nutrients and oxygen, triggering angiogenesis that plays a critical role in tumor growth, progression and metastasis. Consequently, immense focus has converged onto inhibitors of angiogenesis in cancer treatment, such as Nintedanib, which has shown exceptional antitumor activity *via* inhibiting cell proliferation and the resulting tumor growth, primarily due to its combined action on tumor cells, endothelial cells and pericytes. Accordingly, here we assessed both *in vitro* and *in vivo* efficacy of Nintedanib in PCa. The results showed that Nintedanib decreased cell viability in both androgen dependent- and -independent PCa cells, together with a decrease in cell motility and invasiveness. Nintedanib also reduced the expression of significant genes responsible for cell cycle progression. PCa PC3 xenograft-carrying nude mice treated with Nintedanib showed significantly decreased tumor volume and cell proliferation alongside diminished levels of pro-angiogenic molecules and blood vessel densities. In conclusion, we report that Nintedanib has strong efficacy against PCa in pre-clinical models *via* modulation of various pathways, and that it could be employed as a promising new strategy to manage PCa clinically.

## Introduction

Prostate cancer (PCa) is the most common type of cancer in men; according to Siegel (2017) 161,360 new cases of the disease were estimated for 2017 within the United States alone, with approximately 26,730 resulting fatalities, making PCa the second-largest cause of cancer-associated deaths in the US males^1^. It is estimated that more than 40 million men in the US have undetected PCa so far^2^. The early detection for this type of cancer is particularly crucial; once the disease is discovered locally/regionally, the survival outcome approaches 100% for the 5-year survival rate^3^.

Genetic changes capable of deregulating homeostasis between the epithelial and stromal compartments of the prostate are the main cause of cancer development in this gland^4^. However, the formation of new vessels from pre-existing vessels, namely angiogenesis, also plays a vital role in cell proliferation and tumor growth^5^. The development of vessels around the cancer cells provides them with a constant supply of oxygen and nutrients necessary for their growth, thereby contributing to the metastatic spread of the disease through the dissemination of cancer cells^6,7^. This well-understood process involves several growth factors and their receptors being induced by both, the microenvironment and by the tumor cells, altering the equilibrium between pro- and anti-angiogenic factors^8,9^.

Several tyrosine kinase inhibitors of angiogenesis have been shown to possess anti-tumor activity, such as sorafenib, sunitinib, erlotinib and vandetanib for the treatment of several types of cancers^10–13^. Nevertheless, these agents either fail to show improvements or prove to be excessively toxic at some point along the treatment, even when used in combination with well-established chemotherapeutic agents^14–16^. This failure in improving long-term survival or decreasing cancer recurrence rates after treatment might be partly attributed to the fact that these compounds act through inhibition of a specific pathway involved in angiogenesis, allowing the cancer cells to act *via* alternate signaling mechanisms and their crosstalk, to promote tumor growth^17^. Several studies have shown that after simultaneous inhibition of multiple proangiogenic pathways, there is a significant decrease in tumor angiogenesis^18^. Therefore, major attention has been paid to novel agents such as Nintedanib (BIBF 1120), which is capable of inhibiting all three families of receptors engaged in the process of angiogenesis. This angiokinase inhibitor not only targets VEGFR (vascular endothelial growth factor receptor) involved in both cell proliferation and migration, but also PDGFR (platelet-derived growth factor receptor) and FGFR (fibroblast growth factor receptor), indirectly responsible for providing sustenance to new vessels by controlling the action of pericytes and smooth muscle cells^5,6^.

Nintedanib has shown interesting preliminary results in the treatment of non-small cell lung^19^, salivary gland^20^, ovarian^21^ and hepatocellular carcinomas^22^. Furthermore, Nintedanib has no reported drug-drug interactions when administered along with other chemotherapeutic agents^23^. Importantly, we have previously reported the *in vivo* efficacy of Nintedanib in pre-clinical mouse models of PCa; in that background, the present study was an effort to understand the molecular mechanisms involved in Nintedanib efficacy against PCa by evaluating its effects both *in vitro* and *in vivo* in human PCa cell lines and human PCa tumor xenograft model, respectively.

## Results

### Nintedanib treatment significantly decreased cell viability of both androgen-independent and -dependent human PCa cells

**Figure 1:**
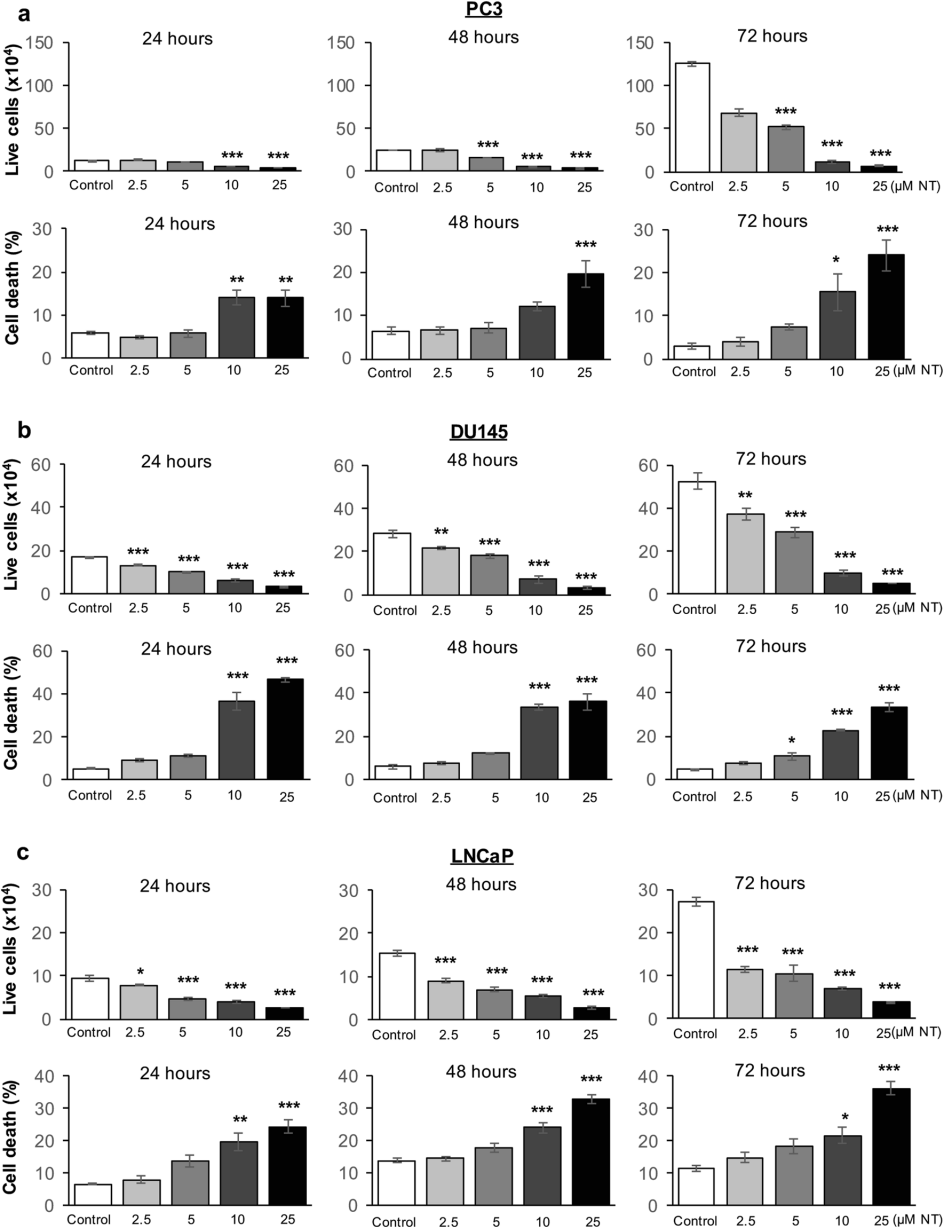
Nintedanib treatment significantly decreased cell viability in both androgen- dependent and -independent human PCa cells. Live cell number and percent of cell death in (**a**) PC3, (**b**) DU145, and (**c**) LNCaP cells after 24, 48 and 72 h of treatment. For live cell number, values are expressed as mean ± S.E.M. (n = 3) and as percentage ± S.E.M for percent of cell death (ANOVA followed by Dunnett’s test), ^*^p < 0.05; ^**^p < 0.01; ^***^p < 0.001.

The trypan blue exclusion assay for cell viability in PC3 cells showed the dose-dependent efficacy of the drug in significantly decreasing the number of live cells and increasing cell death proportional to the drug exposure time. Briefly, at all evaluation time-points (24, 48 and 72 h), there was a significant increase in the percentage of PC3 dead cells after treatment with 10 µM and 25 µM of Nintedanib (Fig. 1a). For DU154 cells, we observed a significant decrease in live cells number within the first 24 h, an effect that sustained after 48 and 72 h at all three doses tested. Also, there was a significant increase in cell death with 10 µM and 25 µM Nintedanib at all time-points (Fig. 1b). The effects of Nintedanib on LNCaP cells were similar to those observed for PC3 and DU145 cells; higher concentrations of the drug were able to significantly increase cell death and decrease live cell number after 24, 48 and 72 h of exposure (Fig. 1c).

### Nintedanib significantly reduced colony formation potential of human PCa cells

**Figure 2:**
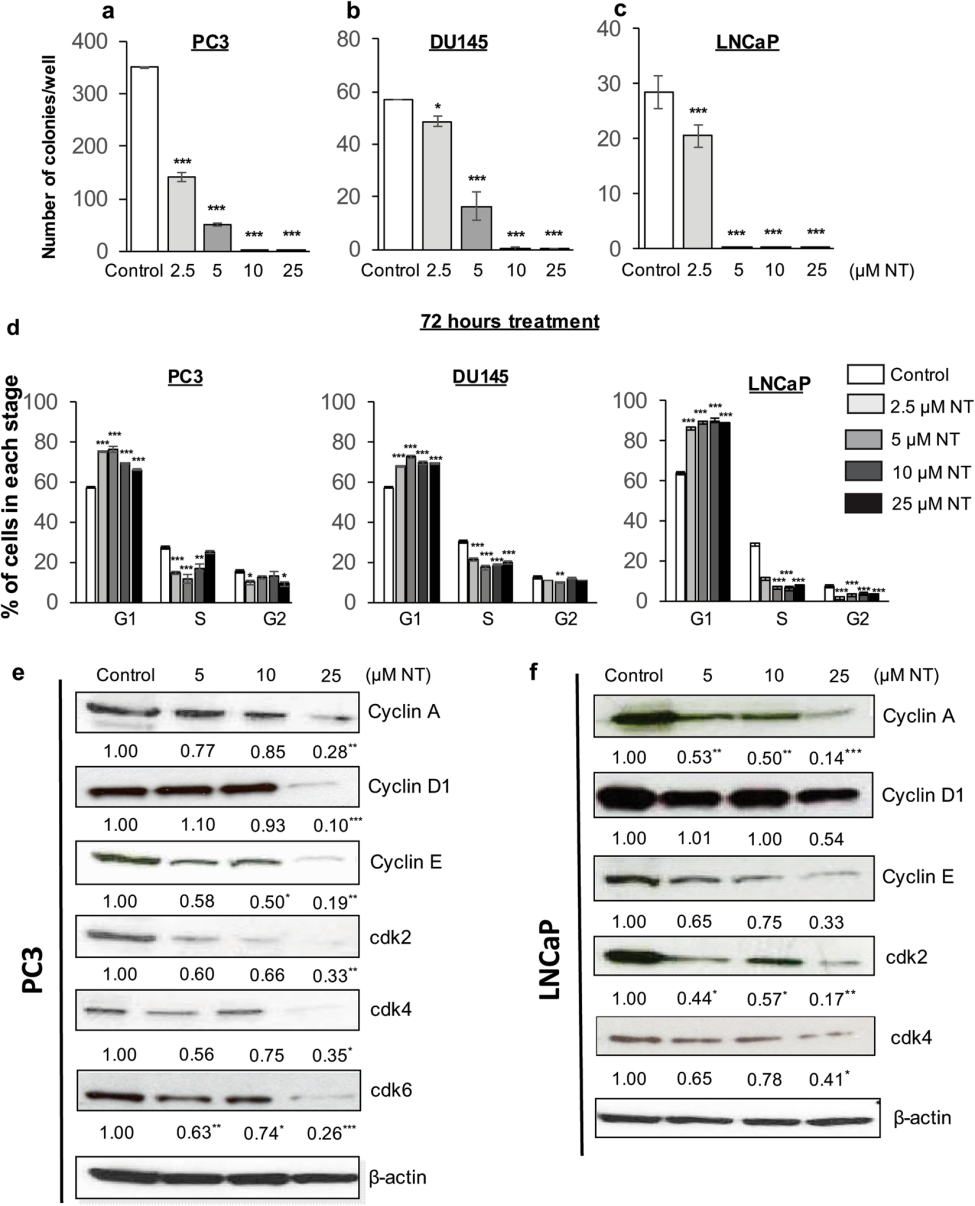
Nintedanib significantly reduced the colony formation ability of human PCa cells and led to their arrest in G1-phase of cell cycle. Number of colonies formed in (**a**) PC3, (**b**) DU145, and (**c**) LNCaP cells after 48 h of Nintedanib exposure. Values are expressed as mean ± S.E.M. (n = 3). ANOVA followed by Dunnett’s test; ^*^p < 0.05; ^***^p < 0.001 for number of colonies generated. (**d**) Percentage of PC3, DU145 and LNCaP cells in different stages of cell cycle after 72 h of treatment with different doses of Nintedanib. Values are expressed as mean ± S.E.M. (n = 3). ANOVA followed by Dunnett’s test; ^*^p < 0.05; ^**^p < 0.01; ^***^p < 0.001. Western blot based detection of cell cycle associated molecules viz., Cyclins A, D1 and E, and CDKs 2, 4 and 6 in (**e**) PC3, and (**f**) LNCaP cells after 72 hours of Nintedanib treatment. Densitometric assessments are reported as mean values relative to β-actin band intensity ANOVA followed by Dunnett’s test; ^*^p < 0.05; ^**^p < 0.01; ^***^p < 0.001.

In colony formation assay, Nintedanib significantly reduced the number of colonies (>50 cells) formed by PC3 cells at 2.5 and 5.0 µM doses. Additionally, exposure to 10 and 25 µM of Nintedanib completely prevented any colony formation (Fig. 2a). In DU145, exposure with 2.5 and 5.0 µM Nintedanib also caused a significant reduction in the number of colonies formed. Furthermore, the colony formation was completely prevented after treatment with higher doses (10 and 25 µM) (Fig. 2b). For LNCaP cells, 2.5 µM of Nintedanib strongly reduced the colony formation, while no colonies were formed after exposure to 5.0, 10 and 25 µM of Nintedanib (Fig. 2c).

### Nintedanib exposure led to arrest of human PCa cells in G1-phase by reducing protein levels and gene expression of key molecules involved in cell cycle progression

Cell cycle analysis by flow cytometry showed that Nintedanib treatment at all doses resulted in a significant arrest of cells in G1-phase in case of all human PCa cell lines, namely PC3, DU145, and LNCaP cells (Fig. 2d). Since effects were comparable in all cells lines, molecular analysis were done only in PC3 and LNCaP cell lines representing androgen-independent and -dependent PCa, respectively. In case of PC3 cells, the levels of key molecules involved in cell cycle progression such as Cyclin A, D1 and E as well as CDKs 2, 4 and 6 were significantly reduced dose-dependently after 72 h treatment with Nintedanib (Fig. 2e). Significantly decreased levels of Cyclin A and E and CDKs 2 and 4 were also observed after treatment with all doses in LNCaP cells, besides significant decrease in Cyclin D1 after treatment with 25 µM dose of Nintedanib (Fig. 2f).

**Figure 3:**
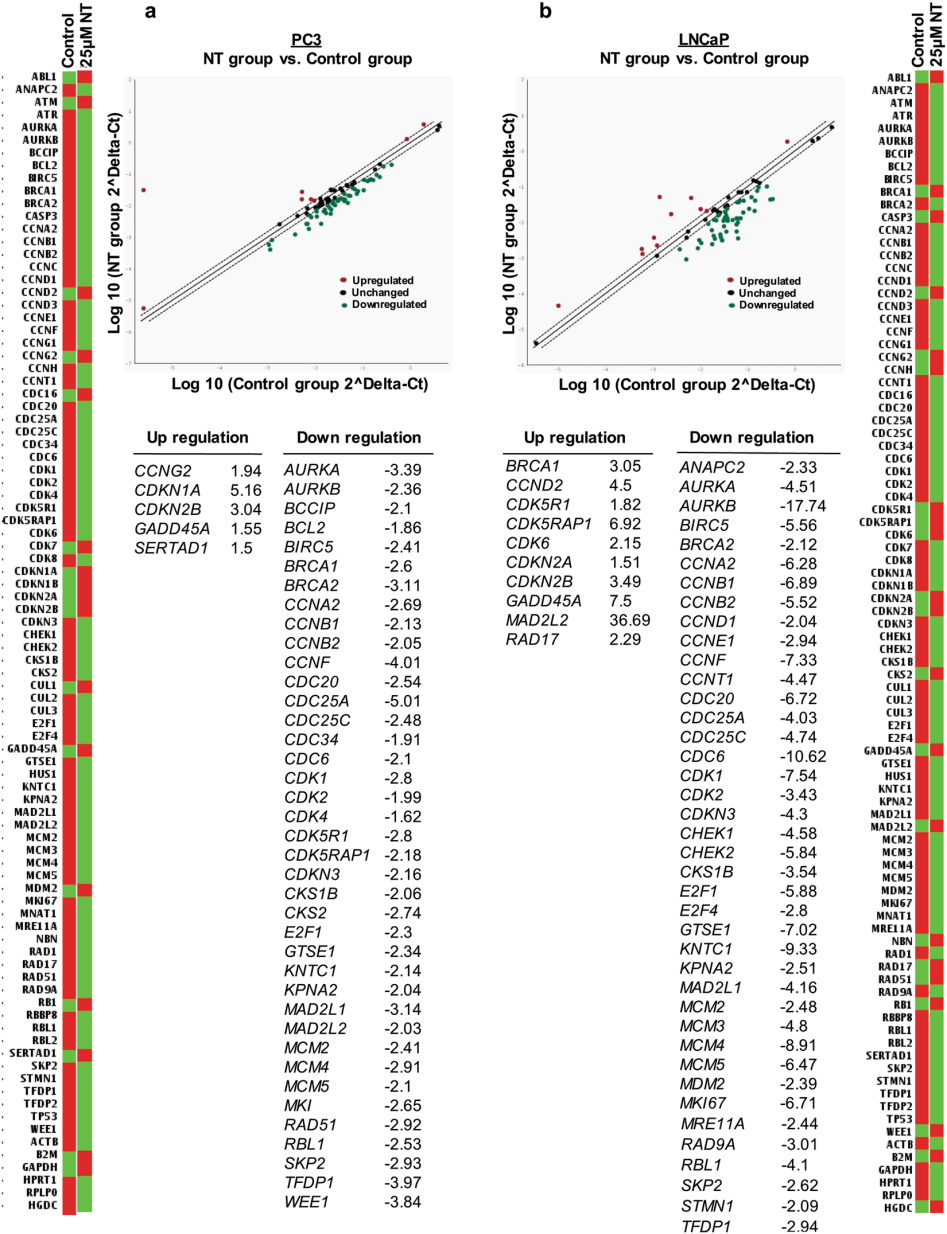
Nintedanib treatment altered the gene expression of cell cycle molecules in human PCa cells after 72 h of treatment. RT^2^qPCR analysis using Human Cell Cycle RT^2^ profiler^TM^ PCR Array (Qiagen) to assess the effect of Nintedanib on expression of genes associated with cell cycle regulation in (**a**) PC3, and (**b**) LNCaP cells. The relative quantification of gene expression between control and Nintedanib treated samples was achieved by normalization against endogenous *GAPDH* and *β-Actin* using the ΔΔCT method of quantification and the data was analyzed using the software provided by the manufacturer. The list details the up and downregulated genes in PCa cells after Nintedanib treatment.

Based on these observations, we further investigated the effects of Nintedanib on the expression of significant genes related to cell cycle progression. The RT^2^qPCR human cell cycle array showed an altered expression of 44 genes in PC3 cells and 50 genes in LNCaP cells after 72 h of Nintedanib exposure (25 µM) (Fig. 3). Notably, in PC3 cells, treatment with the drug upregulated the expression of genes involved in cell cycle arrest such as *CCNG2*, *CDKN1A*, *CDKN2B and GADD45A* (Fig. 3a). Furthermore, Nintedanib treatment downregulated the expression of important genes responsible for G1 phase transition such as *CDC25A*, *CDC34*, *CDK4* and *CDKN3* as well as 16 genes involved in positive regulation of cell cycle such as *AURKA*, *CCNA2*, *CCNB1*, *CCNB2*, *CCNF*, *CDC20*, *CDC25C*, *CDC6*, *CDK1*, *CDK2*, *CDK5R1*, *CKS1B*, *E2F1*, *KNTC1*, *SKP2* and *TFDP1*. Treatment of PC3 cells with Nintedanib also led to a downregulation of genes responsible for DNA synthesis and replication such as *MCM2*, *MCM4*, *MCM5* and *WEE1* (Fig. 3a).

As for LNCaP cells, Nintedanib exposure upregulated genes involved in cell cycle arrest such as *BRCA1*, *CDKN2A*, *CDKN2B*, *GADD45A*, *MAD2L2*, and *RAD17* (Fig. 3b). Importantly, LNCaP exposure to the drug downregulated 17 important genes involved in positive regulation of cell cycle such as *AURKA*, *BRCA2*, *CCNB1*, *CCNB2*, *CCNF*, *CCNT1*, *CDC20*, *CDC25C*, *CDC6*, *CDK1*, *CDK2*, *CKS1B*, *E2F4*, *KNTC1*, *MKI67*, *RAD9A*, and *TFDP1*. In addition, cells treated with Nintedanib displayed downregulated genes involved in G1 phase transition such as *ANAPC2*, *CCND1*, *CCNE1*, *CDC25A*, *CDKN3*, *E2F1* and *SKP2*, besides *MCM2*, *MCM3*, *MCM4* and *MCM5*, responsible for DNA synthesis and replication (Fig. 3b).

### Nintedanib inhibited the migratory potential of human PCa cells

**Figure 4:**
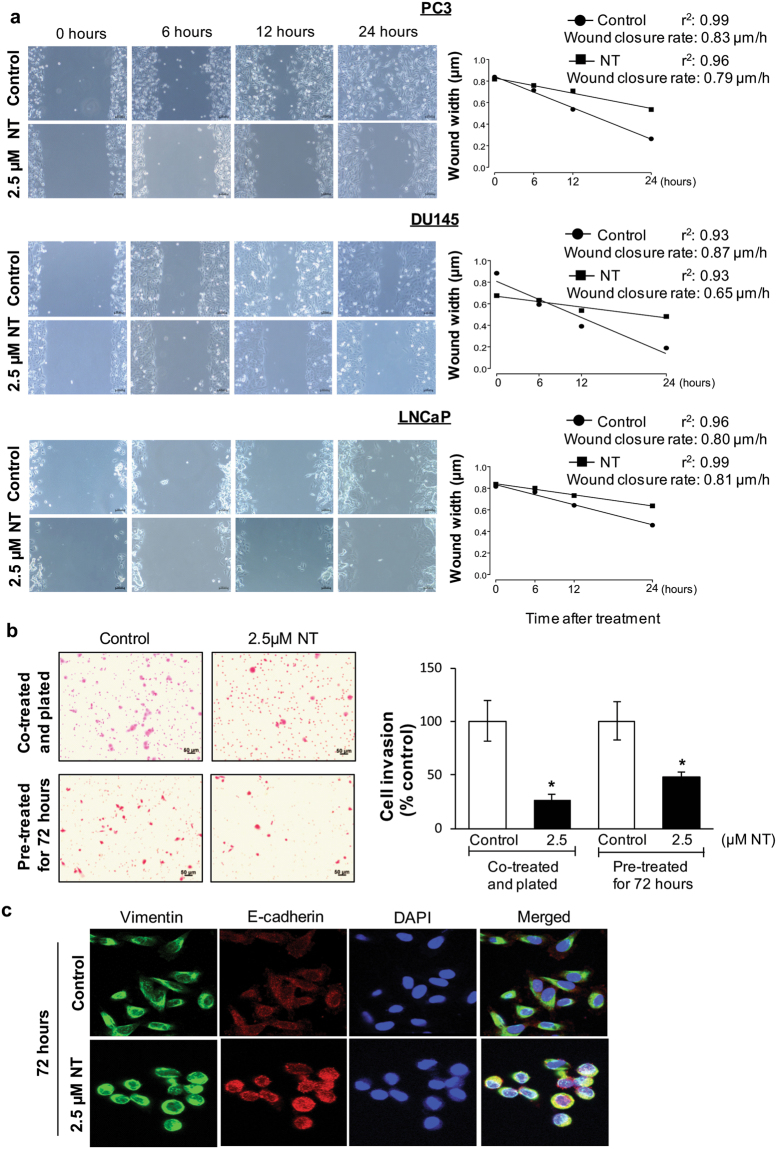
Nintedanib decreased migratory and invasive potential of human PCa cells. (**a**) Wound assay with representative images of wound width over time and wound closure rate for PC3, DU145 and LNCaP cells. Bars = 100 µm. A scatter plot shows the wound width over time, and a linear regression was run to obtain r^2^. (**b**) Cell invasion assay using PC3 cells exposed to DMSO or Nintedanib 2.5 µM during invasion assay, or pre-treated for 72 h, washed and then equal number seeded for invasion assay. Values are expressed as mean ± S.E.M. (n = 3) (two-tailed t-test between treated groups and its respective control). ^*^p < 0.05. Bars = 50 µm. (**c**) Immunoflouresence staining for Vimentin (green) and E-cadherin (red) in DMSO control and 2.5 µM Nintedanib treated PC3 cells after 72 h of incubation. Representative flourescent pictures are shown at 600X. DAPI: 4′,6-diamidino-2-phenylindole.

Wound closure assay in PC3, DU145, and LNCaP PCa cells was performed in the presence of Nintedanib (Fig. 4a).

Results indicated that the PCa cells treated with 2.5 µM of Nintedanib required longer time to close the wound (significantly decreased wound closure rate) (Fig. 4a), demonstrating that the drug Nintedanib inhibited the migratory potential of PCa cells. Although wound closure was significantly inhibited by the drug within 24 h, PC3 cells (control) showed a more effective wound closure than the ones observed in LNCaP and DU145 due to higher invasiveness of PC3 cells (Fig. 4a).

### Treatment with Nintedanib significantly reduced PC3 cell invasiveness *via* increased expression of epithelial marker E-cadherin

Invasion assay was performed on PC3 cells (since this particular cell line has higher invasive potential) using trans-well chambers with matrigel coated 24-well cell culture inserts (8-μm pores) from BD Biosciences, San Jose, CA^24^. Results indicated that in the study where the cells were seeded in chambers in the presence of DMSO or Nintedanib (2.5 µM) and incubated for 22 h (Fig. 4b), the number of invaded PC3 cells was significantly reduced by 72.4%. On the other hand, in the study where cells were pre-treated for 72 h with the same concentration of Nintedanib and then seeded in trans-well, there was a 52.1% reduction in invaded cells compared to DMSO treated controls (Fig. 4b). Importantly, to delineate whether the inhibitory effect of Nintedanib on the invasive potential of PC3 cells was mediated by its effect on mesenchymal to epithelial transition phase, Nintedanib (2.5 µM) treated PC3 cells were subjected to immunofluorescence staining for mesenchymal marker (Vimentin) and epithelial marker (E-cadherin). Results indicated that Nintedanib treatment caused an increase in E-cadherin expression in PC3 cells without affecting Vimentin levels after 72 h of exposure (Fig. 4c); no effect on these markers was observed at earlier time points.

### Nintedanib inhibited the growth of human PCa PC3 tumor xenograft in nude mice

**Figure 5:**
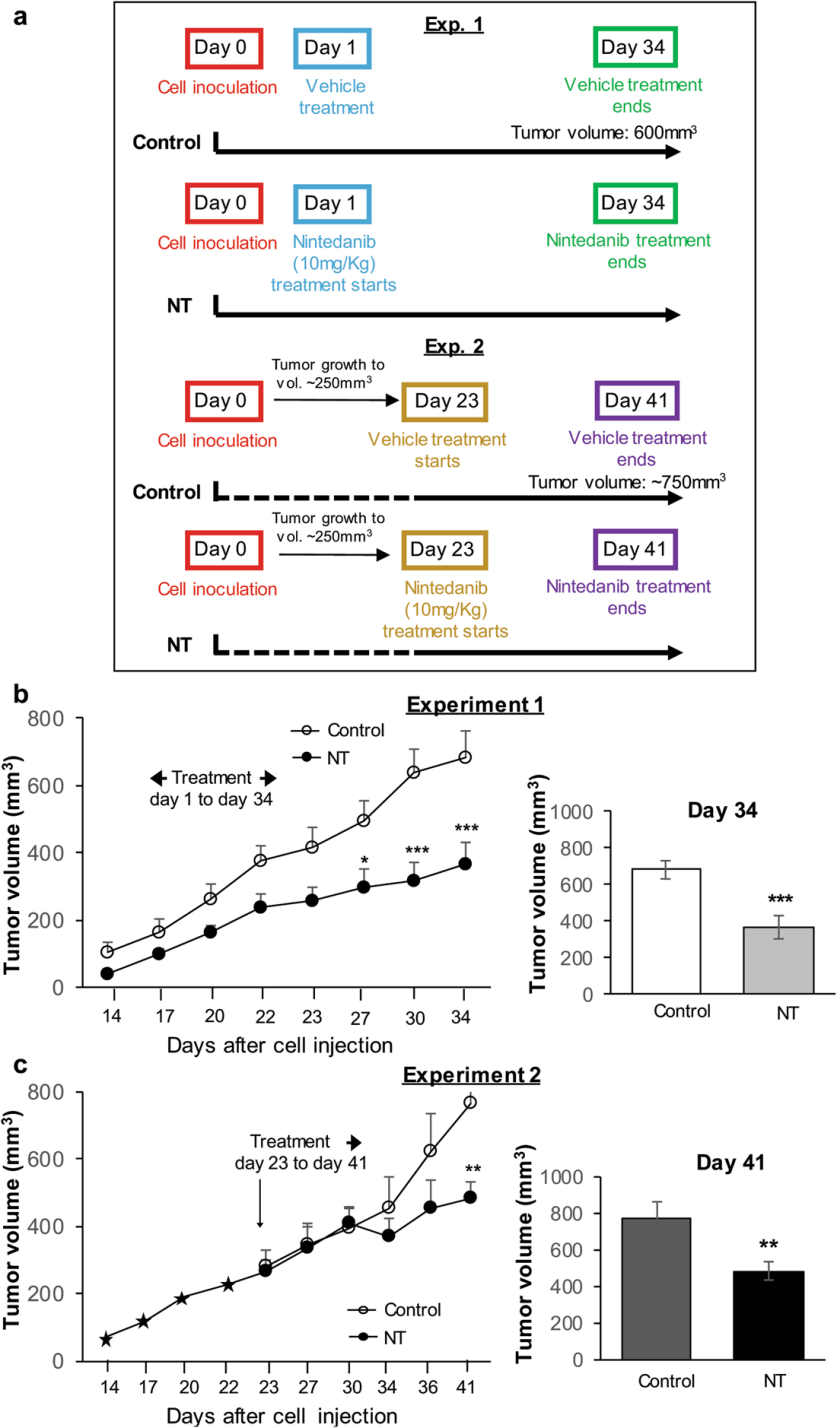
Nintedanib inhibited the growth of human PCa PC3 tumor xenograft in nude mice. (**a**) Schematic plan detailing the Experimental 1 and 2 strategies employed for performing Nintedanib efficacy study against PC3 tumor xenografts in immunocompromised mice. Experiment 1 (animals treated from day 1 after cell injection till study end), and Experiment 2 (animals with established tumors treated from day 23 after cell injection till study end). (**b**–**c**) Tumor volume as a function of time (left panel) and at the study end (right panel) for Experiment 1and Experiment 2. Values are expressed as mean ± S.E.M. (n = 5–8 mice/group) (two-tailed t-test between treated groups and its respective control). ^*^p < 0.05; ^**^p < 0.01; ^***^p < 0.001.

Next, to determine whether the anti-PCa effects of Nintedanib could also be observed under *in vivo* conditions, we performed pre-clinical efficacy studies against human PCa PC3 xenograft tumors using immunocompromised mice. All animal experiments were performed according to the IACUC-approved animal protocol from the University of Colorado Denver. Athymic nude mice (Crl:NU(NCr)-Foxn1^nu^) were purchased from Charles River Laboratories and housed at the animal facility at University of Colorado (Anschutz Medical Campus) for 1 week for acclimatization and fed AIN-76A diet. To determine the effect of Nintedanib treatment on prostate tumor growth, PC3 tumor xenografts were grown subcutaneously (s.c.) in nude mice. About 2 × 10^6^ PC3 cells were suspended in 50 µL of serum-free medium (RPMI), mixed with 50 µL of matrigel and injected s.c. in the right flank of each mouse to initiate tumor growth. 24 h after injection, mice were randomly divided into two experiments. In Experiment 1 (Fig. 5a), mice were orally gavaged (100 µl) with either vehicle alone (Control group) or Nintedanib (NT, 10 mg/Kg body weight/day, 5 days/week in 10% v/v Tween 20 as vehicle) 24 h following cells injection, and treatments continued till study end. Animals in this experimental group were sacrificed (after 34 days of treatment initiation) when the average tumor volume in control group reached 600 mm^3^ (study end time). In Experiment 2, the tumors were allowed to grow to ~250 mm^3^ (till day 23) and then the animals were randomly divided in two groups and respective treatments (either vehicle-alone or Nintedanib) initiated; oral dosing and daily treatment schedule were similar to Experiment 1. Treatments were continued till study end (until the tumor volume reached 750 mm^3^ in the vehicle control group) which was approx. after 19 days of treatment initiation of established tumors (Fig. 5a). In both the experimental strategies, throughout the study, body weight and tumor volume were recorded biweekly. The tumor volume was measured using a digital caliper and calculated using the formula 0.5236 L_1_ (L_2_)^2^, where L_1_ is the long axis and L^2^ is the short axis of the tumor. At the termination of each study, mice were weighed and euthanized; tumors were excised, measured and weighed. Part of the tumor was fixed in 10% phosphate-buffered formalin for immunohistochemical (IHC) analyses and another part of tumor tissue was snap frozen in liquid nitrogen and stored at −80 °C for immunoblot analyses. The body weights (corrected for tumor weights at study end), weights of liver, spleen and genitourinary tract did not differ considerably between study groups.

In Experiment 1, animals treated with Nintedanib 24 h after tumor cell injection till study end (from day 1 to 34) showed a strong decrease in tumor volume in a time-dependent manner throughout the treatment, compared to vehicle control group (Fig. 5b, left panel). Finally, at study end (day 34) the tumor volume in Nintedanib group decreased by ~48%, P <0.001 (Fig. 5b, right panel). Notably, in Experiment 2, when the animals with established tumors received Nintedanib from day 23 onwards, the tumor volume starting decreasing after ten days into Nintedanib treatment (Fig. 5c, left panel), and by the end of study (day 41) there was a significant decrease (~37%, P < 0.01) in tumor volume compared to vehicle control group (Fig. 5c. right panel). In addition, a substantial decrease (though not significant) was observed in tumor weights of treated groups in both experimental studies (data not shown).

### Differential inhibitory effect of Nintedanib treatment on tumor cell proliferation and angiogenesis is dependent on tumor stage

**Figure 6:**
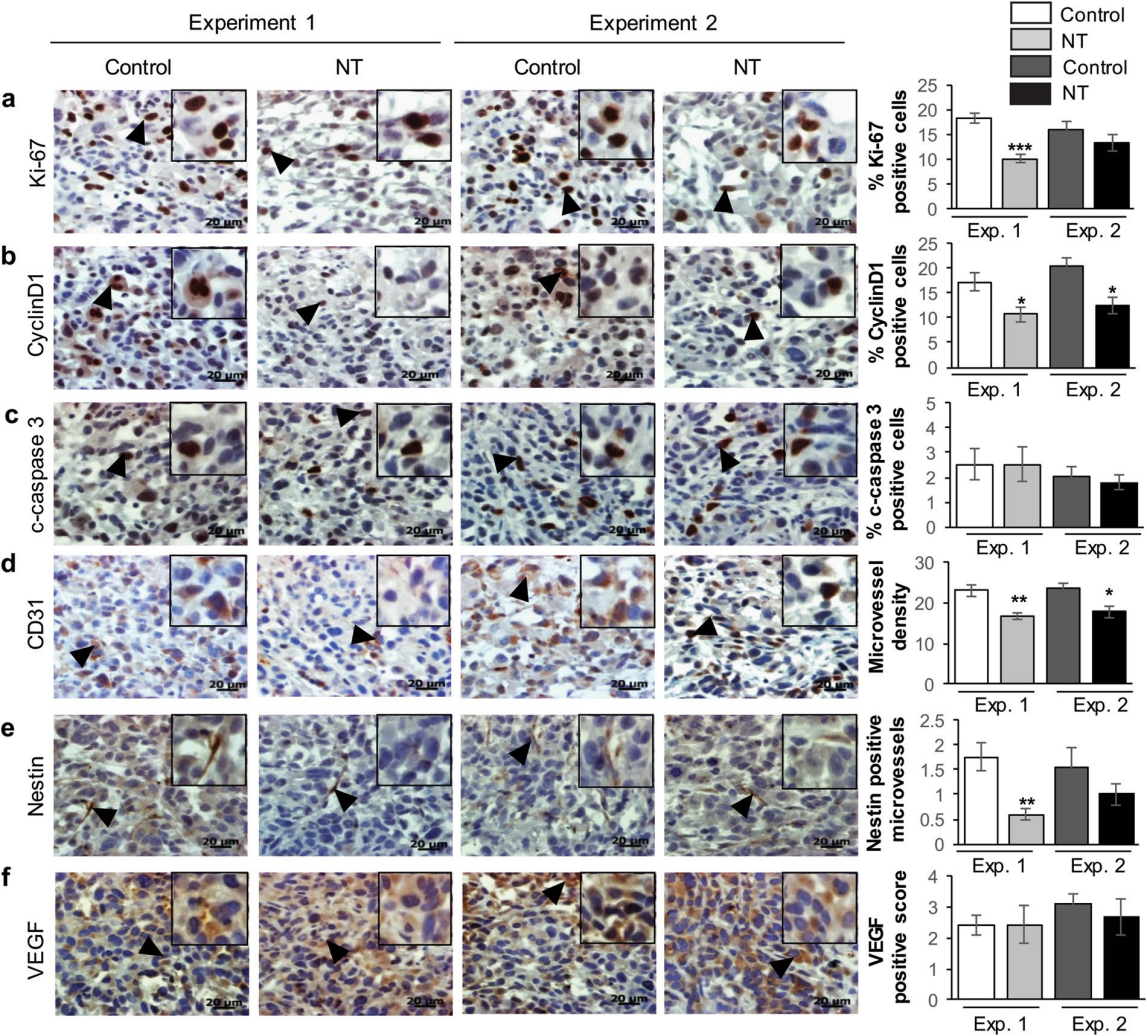
Nintedanib treatment decreased cell proliferation and microvessel density in PC3 xenograft tumors. Immunohistochemical staining of PC3 tumors for (**a**) Ki-67, (**b**) CyclinD1, (**c**) cleaved-caspase 3 (c-caspase 3), (**d**) CD31 (microvessel density), (**e**) Nestin, and (**f**) VEGF expression. Data details the comparative outcomes from both Experiment 1 and Experiment 2 study designs relative to vehicle controls. Values are expressed as % positive cells or as immunoreactivity score (arbitrary vales) as detailed in Methods section. Positively stained markers are shown by arrowhead (►). Representative photographs are presented at 400X; inset represents further magnification of a part of the photograph. Values are expressed as mean ± S.E.M. (n = 5) (two-tailed t-test between treated groups and its respective control). ^*^p < 0.05; ^**^p < 0.01; ^***^p < 0.001.

To determine whether different mechanisms were involved in Nintedanib efficacy against PCa tumor growth, when it was administered during different stages of tumor growth, we performed extensive molecular analysis of the tumor tissues from both xenograft studies detailed above (Fig. 5). Immunohistochemical analysis of tumor tissues for proliferation marker Ki-67 revealed that tumor cell proliferation was significantly decreased by Nintedanib compared to vehicle controls when the mice were exposed to the drug early-on, as in Experiment 1; though there was some decrease in tumor proliferation by Nintedanib in Experiment 2 as well, but it was not statistically significant (Fig. 6a). Interestingly, a significant decrease in Cyclin D1 positive cells was observed in Nintedanib groups (from both studies) when compared to its respective control groups (Fig. 6b). While decreasing proliferative index was identified as a factor that could be associated with decreased tumor volumes in Nintedanib group; induction of cell death by apoptosis was ruled out to play any role in Nintedanib anti-tumor effects as evidenced by no effect on cleaved-caspase 3 (c-caspase 3) positive cells in both studies (Fig. 6c). On the other hand, with regard to angiogenesis, it was observed that while Nintedanib treatments in both studies could significantly decrease microvessel density (CD31 immunostaining) (Fig. 6d), the process of neo-angiogenesis (new formation of small microvessels) as identified by positive staining for Nestin was significantly decreased by Nintedanib only in those tumors that were treated early on after injecting PC3 cells (Fig. 6e). Interestingly, there was no significant difference in VEGF expression by Nintedanib compared to their respective vehicle controls in both studies (Fig. 6f).

**Figure 7:**
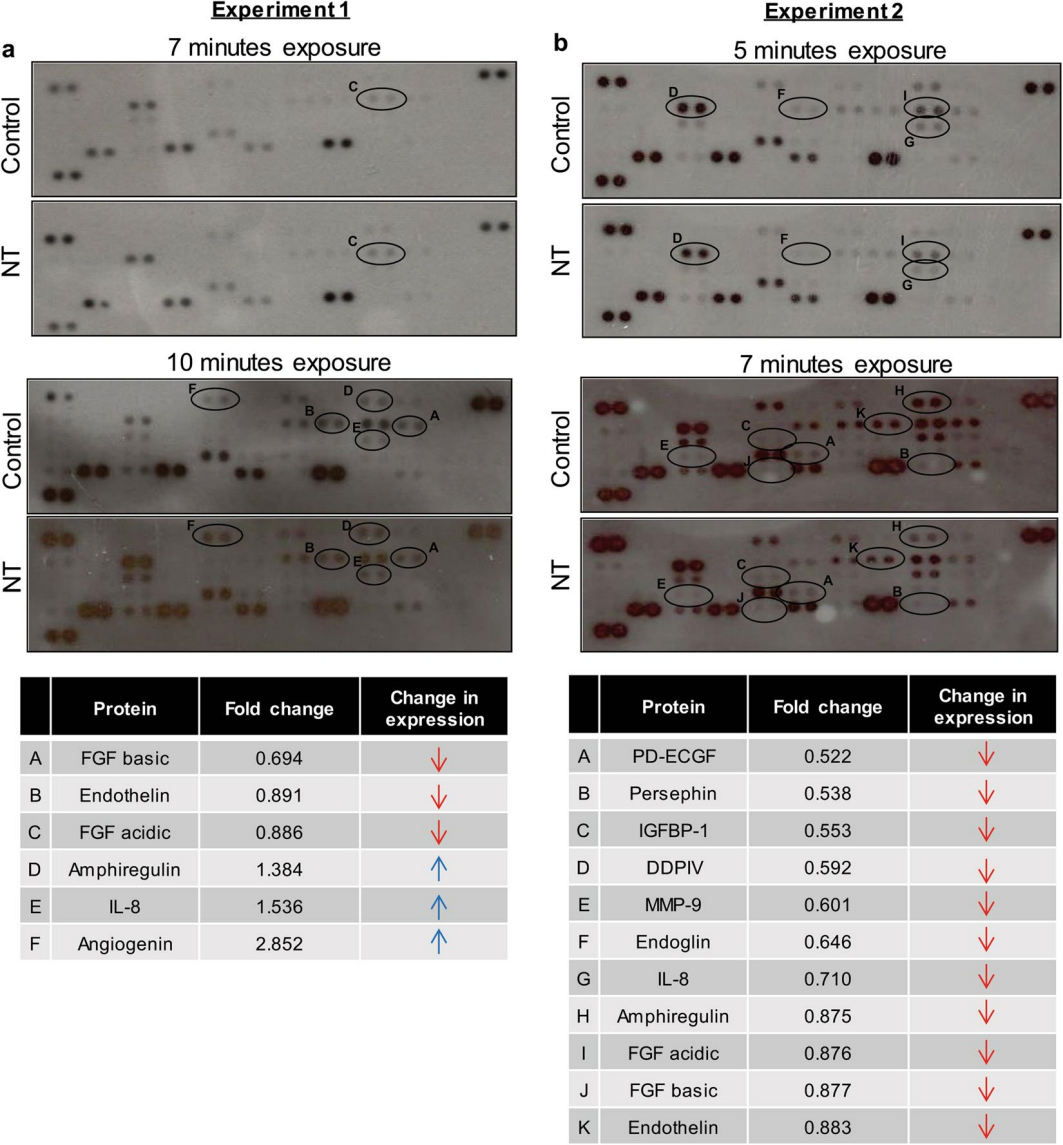
Inhibitory effect of Nintedanib treatment on the protein expression of pro-angiogenic molecules in tumor tissues is dependent on tumor stage. Comparative outcomes of Human Angiogenesis Proteome Profiler™ Array analysis of PC3 tumor xenograft tissues from both Experiment 1 and Experiment 2 studies. Images represent the protein blot profile of representative tumor sample lysates (2 dot lanes per molecule). Molecules showing difference in densitometric values between vehicle control and Nintedanib groups are marked by a circle. Fold changes of down regulated molecules (red arrow) and upregulated molecules (blue arrow) are listed for both studies.

To confirm the antiangiogenic effects of Nintedanib treatment *in vivo*, tissue lysates from both studies were subjected to human angiogenesis profiler array. The results showed reduced protein levels of pro-angiogenic molecules such as FGF acidic, FGF basic and Endothelin, but increased levels of Amphiregulin, Interleukin 8 (IL-8), and Angiogenin in Nintedanib-treated tumors, compared to controls, in Experiment 1 (Fig. 7a). In Experiment 2, there was an overall decrease in the levels of most of the pro-angiogenic molecules such as platelet-derived endothelial cell growth factor (PD-ECGF), Persephin, Insulin-like growth factor-binding protein 1 (IGFBP-1), Dipeptidyl peptidase-4 (DDPIV), Endoglin, Matrix metallopeptidase 9 (MMP-9), IL-8, Amphiregulin, FGF acidic, FGF basic, and Endothelin in Nintedanib-treated tumors compared to controls (Fig. 7b).

## Discussion

The rationale for the present study was to further understand the mechanistic pathway by which Nintedanib inhibits PCa growth progression, since we have previously shown that this drug delays tumor development in a pre-clinical (transgenic) mouse model of PCa^25^. Herein, we have demonstrated that exposure to the drug decreased cell viability and clonogenic capacity, besides increasing E-cadherin expression, which led to a reduced motility and invasiveness in PC3 cells. Nintedanib exposure also led to cell cycle arrest of PCa cells by decreasing gene expression and levels of key molecules involved in cell cycle progression, showing a completely novel mode of action of this antiangiogenic drug. Importantly, immunocompromised mice bearing human PC3 tumor xenografts treated with Nintedanib showed significantly decreased tumor volumes and cell proliferation in addition to diminished levels of key angiogenic molecules.

The results presented in this study have revealed the possible mechanisms by which Nintedanib causes tumor growth inhibition. The observed effects include decreased proliferative index, which leads to a decrease in tumor cell progenies and stalled tumor progression in xenograft mouse model. These effects were attributed to the ability of Nintedanib to affect the expression of key genes pertinent to cell cycle progression and cell cycle arrest, leading the tumor cells to escape the normal cell cycle regulation and preventing uncontrolled cell proliferation of both androgen-dependent and -independent PCa cells^26^. In Nintedanib exposed cells, the downregulation of major cell cycle checkpoint regulatory proteins such as CDKs 2, 4 and 6 and their positive regulators such as Cyclins D1, E and A, besides cell division cycle (CDC) 20 and 25 A led to cell cycle arrest in G1 phase by delaying the cellular entry into the S and G2/M phases of cell cycle^27^. Additionally, the observed downregulation of the transcription factors E2F family members by Nintedanib exposure could also prevent the expression of crucial genes related to DNA synthesis in the cancer cell^28^. Furthermore, Nintedanib exposure upregulated the cyclin-dependent kinase inhibitors such as CDKN1 and 2 that act like negative regulators of cell cycle progression alongside growth arrest and DNA damage-inducible 45 (GADD45), a tumor suppressor known to play a role in mediating the anti-cancer activity of several chemotherapeutic drugs^29,30^. Nintedanib effect leading to cell cycle arrest was confirmed *in vivo*, as shown by decreased Cyclin D1 levels and proliferation rate of tumor cells in PC3 xenografts, which contributes to reduced tumor volume in animals that received the drug.

More than 90% of PCa associated patient fatality is related to the ability of tumor to spread throughout the body^31,32^. Modification in cell structure leading to metastasis; epithelial to mesenchymal transition (EMT), is characterized by loss of cell-cell adhesion and invasion of basement membranes; therefore, substances that promote E-cadherin expression, such as Nintedanib, are expected to prevent EMT^33,34^.

Indeed, the decreased rate of invasion in PC3 cells after Nintedanib treatment presented in this study was accompanied by an upregulation of E-cadherin expression in PCa cells exposed to the drug. It is noteworthy that Nintedanib exposure also exhibited an anti-migratory/-invasive effect in a highly metastatic PCa cell line such as PC3. Zhou *et al*. (2009) have previously shown that overexpression of E-cadherin inhibited invasion in PC3 cells whereas the knockdown of this protein was able to restore invasiveness potential in this cell line, corroborating our results^35^.

Tumor neovascularization/angiogenesis, has an important role in cancer progression, therefore, cancer treatment by anti-angiogenic drugs has been shown to complement conventional therapy since 1971^36^, considering that blocking new vessel formation in the tumor suppresses cancer development and progression^31^. In the present study, we have demonstrated the efficacy of Nintedanib in decreasing angiogenesis in xenograft tumors, since microvessel density measured by CD31 immunostaining in the tumors was significantly reduced in both experiments, concluding that not only was the drug highly effective when administered in early stages of tumor development, but also when angiogenesis was already established. Microvessel density, often assessed by CD31 immunostaining of pre-existing and large sized blood vessels around the tumor, is a hallmark of angiogenesis and a prognostic marker for tumor progression^37^. Additionally, a more sensitive analysis of microvessel density was performed by immunostaining for Nestin; its expression has been correlated with worse prognosis in PCa^38^. We observed that animals receiving Nintedanib in early stages of tumor growth displayed a significant decrease in the newly formed vessel density, although animals treated with the drug later in angiogenesis also displayed a substantial decrease in Nestin expression.

Regarding angiogenesis, the secretion of pro-angiogenic factors that induce new blood vessels for tumor growth and expansion, takes place when tumors reach a size of 1–2 mm^39^. That explains why Nintedanib is more effective in decreasing levels of important pro-angiogenic molecules within the tumor tissues of animals receiving the drug after establishment of angiogenesis, i.e., when angiogenic factors are already secreted by the tumor cells. In conjunction with decreasing levels of both acidic and basic FGF and PD-ECGF, treatment with the drug reduced endothelial cell proliferation and survival besides affecting wound healing, being importantly involved in vasculogenesis and angiogenesis signaling^40^. Furthermore, after Nintedanib treatment, we observed decreased levels of Amphiregulin and Endoglin, which are oncogenic factors that stimulate cellular invasion and motility^41^ and found to be upregulated in the endothelia of newly formed vessels^42^; decreased Angiogenin levels were also observed in Nintedanib tumors^43^. Importantly, Nintedanib also decreased Endothelin levels, a protein secreted by endothelial and epithelial tumor cells that has been shown to activate hypoxia-response under normoxic condition and upregulating matrix metalloproteinases (MMPs), increasing cancer aggressiveness^44^. There is a possibility that by decreasing Endothelin levels, Nintedanib was also effective in decreasing MMP-9 expression, which could possibly lead to decreased tumor invasiveness and metastasis^45,46^.

In summary, this study has presented Nintedanib as a promising drug candidate in the treatment of PCa due to its strong action against tumor cells and angiogenesis, leading to a decrease in tumor growth and progression. Overall, the mechanistic studies revealed that the anti-PCa efficacy of Nintedanib was associated with pronounced anti-proliferative effects in early tumor stages and inhibition of neo-angiogenesis, while the drug effects in more established tumors were mostly associated with significant inhibition of pro-angiogenic molecules resulting in decreased angiogenic support essential for tumor sustenance.

## Material and Methods

### Reagents and cell culture

Human PCa cell lines PC3, DU145 and LNCaP were obtained from American Type Culture Collection (Manassas, VA). Cells were cultured in RPMI 1640 with 10% fetal bovine serum (Hyclone, Logan, UT) under standard culture conditions (37 °C, 95% humidified air and 5% CO_2_). Nintedanib was from MedChem Express (CAS 656247-17-5). Mitomycin C was from Sigma-Aldrich.

### Viability assay

PC3, DU145 and LNCaP cells were plated at a density of 5 × 10^3^ cells/cm^2^ under standard culture conditions. After 24 h, cells were treated with either DMSO alone (control) or with different doses of Nintedanib dissolved in DMSO (concentration not exceeding 0.1% in any treatment (v/v)). At the end of treatment, cells were harvested after brief trypsinization, washed with PBS, and then stained with Trypan blue. The total cell number and dead cells (blue stained) were counted under light microscope using a hemocytometer.

### Clonogenic assay

The cells were cultured in 6-well plates (1 × 10^3^ per well for PC3 and DU145 cells, and 2 × 10^3^ per well for LNCaP). After 24 h, the cells were treated with vehicle or Nintedanib (2.5 µM, 5.0 µM, 10 µM and 25 µM) for 48 h. The evaluation period was different for each cell line, based on its ability to form colonies (5 days - 11 days). By the end of treatment period, cells were washed, fixed with formalin 4% and stained with Crystal Violet solution (Sigma-Aldrich). Photomicrographs were captured using canon power shot digital camera and the number of colonies (with >50 cells) was recorded.

### Cell cycle distribution assay

After identical treatments as detailed earlier in above assays, PC3, DU145 and LNCaP cells were collected and processed for cell cycle analysis. Briefly, cells were plated at a density of 5 × 10^3^ cells/cm^2^, treated with vehicle or Nintedanib (2.5 µM, 5.0 µM, 10 µM and 25 µM) for 72 h. At the end of treatment, cells were suspended in 0.5 ml of saponin/PI solution (0.3% saponin (w/v), 25 mg/ml PI (w/v), 0.1 mM ethylenediaminetetraacetic acid and 10 mg/ml RNase A (w/v) in phosphate-buffered saline) and incubated overnight at 4 °C in dark. Cell cycle distribution was analyzed by flow cytometry using fluorescence-activated cell sorting analysis core facility at the University of Colorado Cancer Center.

### Western immunoblotting assay

PCa cells were treated with vehicle (DMSO) or Nintedanib (5.0 µM, 10 µM and 25 µM) for 72 h, collected and lysed in non-denaturing lysis buffer; approximately 50–80 μg of protein lysate from whole-cell extracts was denatured in 5X sample buffer and subjected to sodium dodecyl sulfate–polyacrylamide gel electrophoresis (SDS-PAGE) on Tris–glycine gel. The separated proteins were transferred on to nitrocellulose membrane followed by blocking with 5% non-fat milk powder (w/v) in Tris-buffered saline (10 mM Tris–HCl, pH 7.5, 100 mM NaCl, 0.1% Tween 20) for 1 h at room temperature. Membranes were probed with the following primary antibodies: rabbit polyclonal anti-Cyclin A (sc751), rabbit polyclonal anti-Cyclin E (sc198), rabbit polyclonal anti-Cyclin D1 (sc718), mouse monoclonal anti-CDK2 (sc6248), rabbit polyclonal anti-CDK4 (sc749), and rabbit polyclonal anti-CDK6 (sc177), followed by the appropriate peroxidase-conjugated secondary antibody and visualized by ECL detection system. Membranes were also re-probed with the loading control β-actin (A2228 Sigma-Aldrich). The autoradiograms/bands were scanned with Adobe Photoshop 6.0 (Adobe Systems, San Jose, CA). The intensity of bands was determined by densitometry using the Image J (Image Analysis and Processing in Java) software for image analyses and reported as mean values relative to β-actin band intensity. The full-length blots at different exposures are provided in the Supplementary Fig. 1.

### RT^2^-qPCR array

PC3 and LNCaP cells were treated either with DMSO or Nintedanib (25 µM) for 72 h. Total RNA was harvested from cultured cells and purified with the RNeasy MinElute Cleanup Kit (Qiagen) according to the manufacturer’s instruction. 1 µg total RNA was reversed transcribed into cDNA using the RT^2^ First Strand Kit (Qiagen). The expression of 84 key genes associated with cell cycle regulation was analyzed by using RT² Profiler™ PCR Array Human Cell Cycle (Qiagen). The PCR array was performed on Applied Biosystems® 7500 Cycler and the PCR cycling condition was set as follows: denaturation for 10 min at 95 °C followed by 40 cycles of 15 sec at 95 °C and 1 min at 60 °C. The relative quantification of gene expression between control and Nintedanib-treated samples was achieved by normalization against endogenous GAPDH and β-Actin using the ∆∆CT method of quantification and the data was analyzed using the software provided by the manufacturer.

### Wound healing assay

The wound healing assay was performed in order to evaluate the anti-migratory efficacy of Nintedanib treatment. Mitomycin C, an antibiotic isolated from the broth of Streptomyces caespitosus, shown to have antitumor activity by inhibiting cell proliferation in a dose-dependent manner, was additionally added in the wound healing assay to ensure that the cells filling the wound were the cells involved in migration and not proliferation. The concentration used (0.5 µM of Mitomycin C) was effective in blocking cell proliferation without causing any toxic effects to the cells, according to viability assay performed. Likewise, the dose of Nintedanib (2.5 µM) was selected for performing wound assay as this dose was effective in reducing the number of live cells without increasing the cell death (Fig. 1), a desirable feature for wound healing assay. All cell lines were plated in 6-well plates in a way that, after 12 h, they had grown to full confluence. Then the layer of cells was scraped with a pipette tip to create a wound. Thereafter, cells were washed twice with PBS and treated with vehicle or Nintedanib (2.5 µM) for 12 h or 24 h (for 24 h time-point, the cells were previously treated with Mitomycin C for 2 h). Photomicrographs were taken at 0, 6, 12 and 24 h. The results were shown as wound closure rate over time and a linear regression was run on the wound width data to calculate the r^2^.

### Invasion assay

Invasion assay was performed using matrigel coated trans-well chambers with 24-well cell culture inserts (8-μm pores) from BD Biosciences (San Jose, CA)^24^. First, the bottom chambers were filled with RPMI media (with 10% FBS) and top chambers were seeded with 1 × 10^5^ cells per well in RMPI media (with 0.5% FBS) and treated with DMSO or 2.5 µM Nintedanib (non-cytotoxic dose). In the second experiment, seeded cells in 2D culture dishes were treated with DMSO or Nintedanib (2.5 µM) for 72 h, collected and equal number of live cells (1 × 10^5^ cells) were plated in the upper well of trans-well chamber inserts. After 22 h of seeding in invasion chamber inserts, matrigel invaded cells on the other side of inserts were fixed, counterstained with hematoxylin/eosin and counted. At the end of assay, cells on the top surface of the membrane (non-invasive cells) were scraped with cotton swabs and cells on bottom side of membrane (invasive cells) were fixed with cold methanol, stained with hematoxylin/eosin and mounted. Invasive cells were manually counted at 400X in 5 random fields on each membrane. Images were taken using Cannon Power Shot A640 camera on Zeiss inverted microscope.

### Immunofluorescence assay

The PC3 cells were seeded on cover slips in 6-well plates and incubated with appropriate treatments (vehicle or 2.5 µM Nintedanib). After 12, 24, 48 and 72 h of treatment, cells were fixed with formalin 4%, permeabilized with 0.1% triton-X for 2 h and blocked with 5% BSA. Cells were then incubated with mouse monoclonal anti-E-cadherin (sc-21791) and goat polyclonal anti-Vimentin (sc-7557) overnight at 4 °C. Next day, cells were incubated with respective fluorescence-tagged secondary antibody from Molecular Probes (Eugene, OR) along with 4′,6-diamidino-2-phenylindole (DAPI). Cell images were captured at 600X magnification on a Nikon inverted confocal microscope using appropriate laser wavelengths to detect the fluorescence emissions.

### Immunohistochemistry

Tumor samples were fixed in 10% phosphate-buffered formalin for 24 h and processed conventionally. The paraffin-embedded tumor sections (5 μm-thick) were deparaffinized using xylene and rehydrated in a graded series of ethanol. Antigen retrieval was performed using 10 mM citrate buffer (pH 6.0) in a microwave. Endogenous peroxidase activity was blocked by immersing the sections in 3.0% H_2_O_2_ in methanol (v/v) for 10 min and in CAS block for 45 min. The sections were then incubated overnight at 4 °C with the respective primary antibodies. Following day, the sections were incubated with biotin-conjugated secondary antibodies for 1 h at room temperature. Thereafter, sections were incubated with conjugated horseradish peroxidase-streptavidin for 60 min at room temperature in a humidified chamber. Next, the sections were incubated with DAB for 5 min at room temperature, washed and then counterstained with diluted Harris hematoxylin for 3 min, and rinsed in Scott’s water. Percentage of positive cells was calculated by counting the number of positive stained cells (brown stained) and the total number of cells at ten arbitrarily selected fields from each tumor. For cytoplasmic staining, arbitrary immunoreactivity scores were allotted based on intensity of brown staining as 0 (no staining), 1 (weak staining), 2 (moderate staining), 3 (strong staining), 4 (very strong staining). Images were captured by AxioCam MrC5 camera at 400X magnification.

### Human angiogenesis profiler array

The relative expression levels of 55 angiogenesis-related proteins in tumor tissues were analyzed using a Human Angiogenesis Proteome Profiler™ Array kit from R&D Systems Inc., according to the manufacturer’s protocol. Briefly, tissue lysates were prepared and the protein concentration was determined with Bio-Rad detergent-compatible protein assay kit (Bio-Rad Laboratories), and 100 µg of protein was subjected to proteome profiler array by mixing the samples with a cocktail of biotinylated detection antibodies and then incubated with a nitrocellulose membrane spotted with capture antibodies in duplicate. Streptavidin–HRP and chemiluminescent detection reagents were used to detect the protein antibodies bound to the capture antibody. The mean spot pixel density was quantified using ImageJ Software. The full-length blots at different exposures are provided in Supplementary Fig. 1.

### Statistical analyses

A one-way analysis of variance (ANOVA) followed by Dunnett’s comparison was used for *in vitro* experiments comparing more than two experimental groups. For all *in vivo* and some *in vitro* experiments (the ones comparing two groups), two-tailed t-test was used. The result outcomes in *in vitro* studies were either based on 2-3 independently run studies and are shown as mean ± S.E.M (standard error mean) or as % values relative to untreated controls.

### Data Availability

The datasets generated during and/or analyzed during the current study are available from the corresponding author on reasonable request.

## Electronic supplementary material

Dataset 1
