# Supplementary material for: Nintedanib inhibits growth of human prostate carcinoma cells by modulating both cell cycle and angiogenesis regulators
Source: Sci Rep. 2018 Jun 22;8:9540. doi: 10.1038/s41598-018-27831-1 (PMC6014981; doi:10.1038/s41598-018-27831-1)
Supplement: Supplementary file 1 — Dataset 1 [file 41598_2018_27831_MOESM1_ESM.pdf]

Nintedanib inhibits growth of human prostate carcinoma cells by modulating both cell cycle and angiogenesis regulators

Raquel Frenedoso da Silva[1,3], Deepanshi Dhar[1], Komal Raina[1,2], Dileep Kumar[1], Rama Kant[1], Valeria Helena Alves Cagnon[3], Chapla Agarwal[1,2], Rajesh Agarwal[1,2*]

[1] *Department of Pharmaceutical Sciences, Skaggs School of Pharmacy and Pharmaceutical Sciences,* [2] *University of Colorado Cancer Center, University of Colorado Anschutz Medical Campus, Aurora, Colorado, USA*

[3] *Department of Structural and Functional Biology, Institute of Biology, University of Campinas (UNICAMP), São Paulo, Brazil*

[*]**Corresponding Author**: Dr. Rajesh Agarwal. Department of Pharmaceutical Sciences, Skaggs School of Pharmacy and Pharmaceutical Sciences, University of Colorado Denver, 12850 E. Montview Blvd, C238, Room V20-2118, Aurora, CO 80045. Phone: (303) 724-4055, Fax: (303) 724-7266, E-mail: Rajesh.Agarwal@UCDenver.edu

Running title: Nintedanib is a novel anti-prostate cancer agent.

Keywords: Prostate cancer; Nintedanib; Angiogenesis, Cell Cycle; EMT

The sequence of samples in a lane is as- C, 5, 10, 25 µM NT
‘S’ represents different sample sets

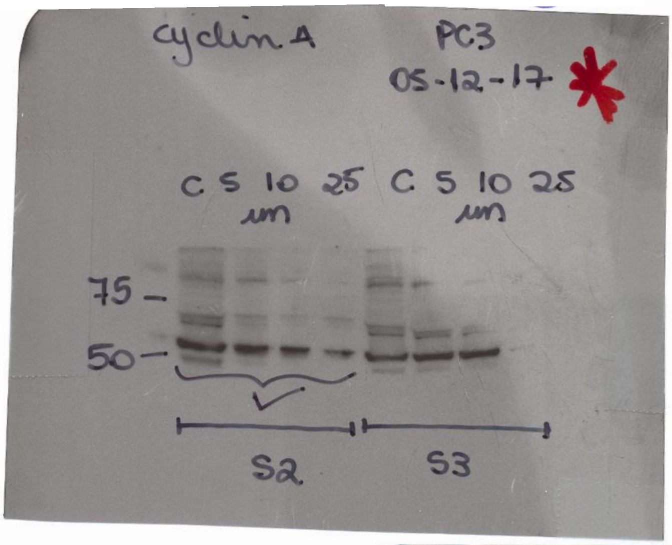

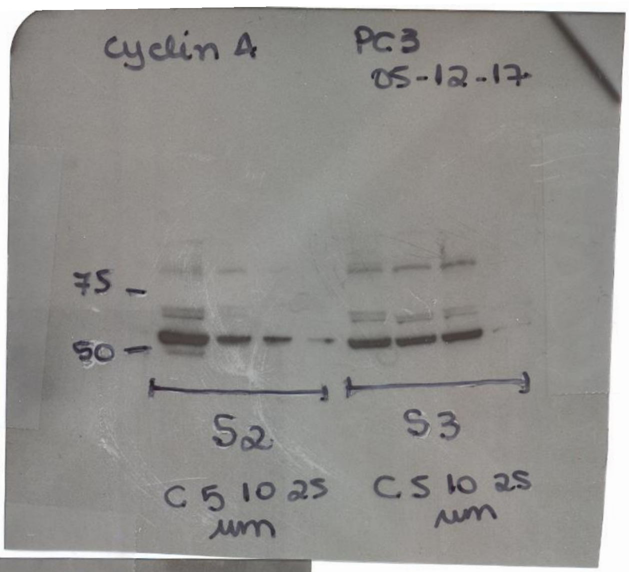

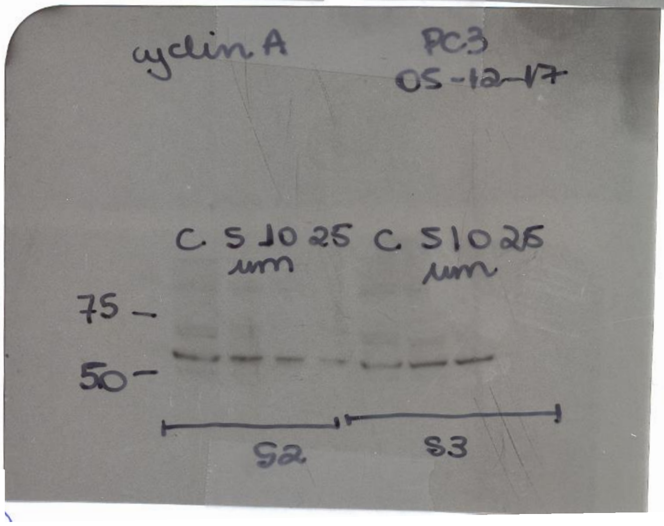

Supplementary information for Figure 2e **PC3** cells- **Cyclin A** (54 KDa)
Blots marked with asterisk (red) were used in main manuscript file

The sequence of samples in a lane is as- C, 5, 10, 25 µM NT
'S' represents a sample set

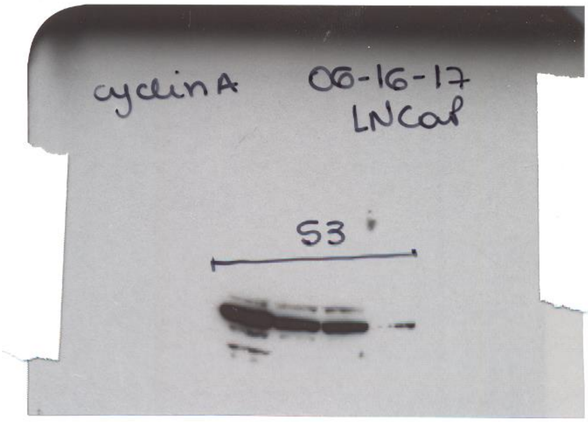

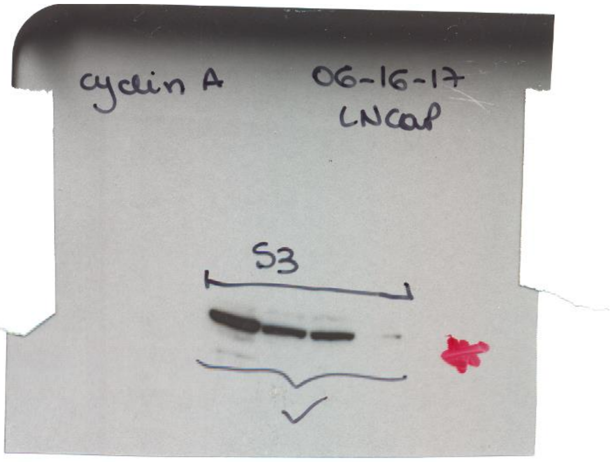

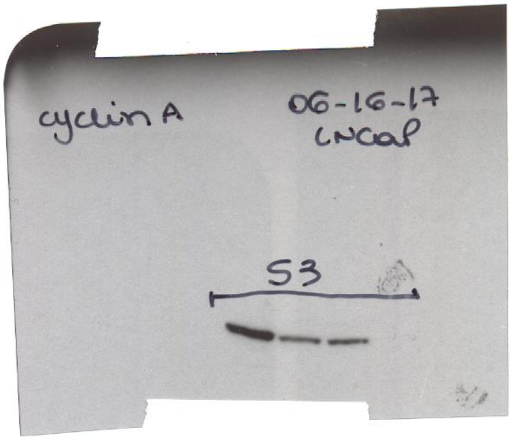

Supplementary information for Figure 2f **LNCaP** cells- **Cyclin A** (54 KDa)
Blots marked with asterisk (red) and checked were used in main manuscript file

The sequence of samples in a lane is as- C, 5, 10, 25 µM NT
'S' represents a sample set

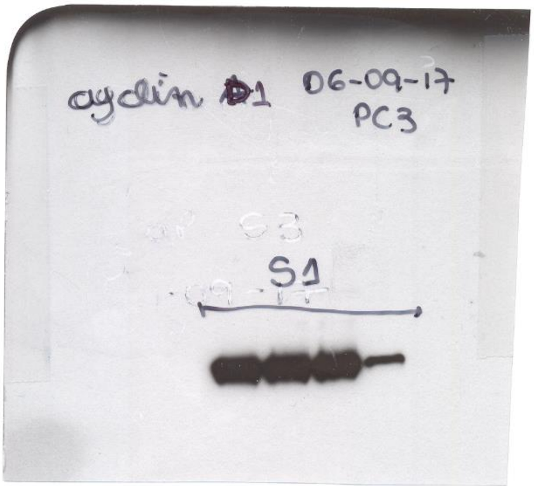

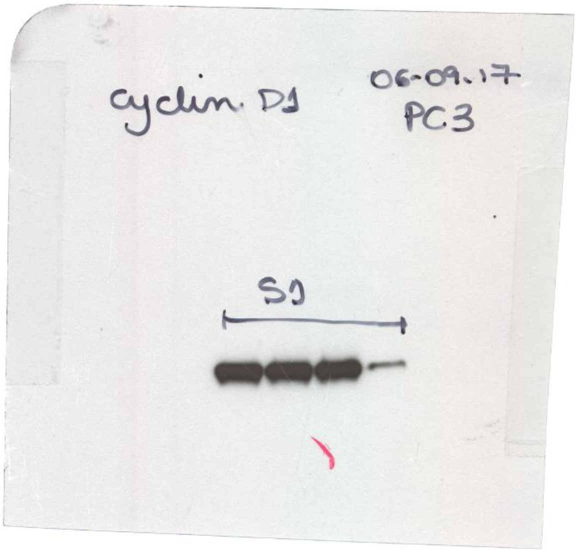

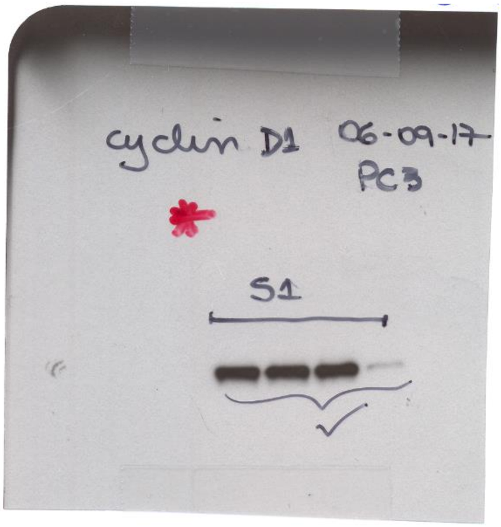

Supplementary information for Figure 2e **PC3** cells- **Cyclin D1** (31 KDa)
Blots marked with asterisk (red) and checked were used in main manuscript file

The sequence of samples in a lane is as- C, 5, 10, 25 µM NT
'S' represents different sample sets

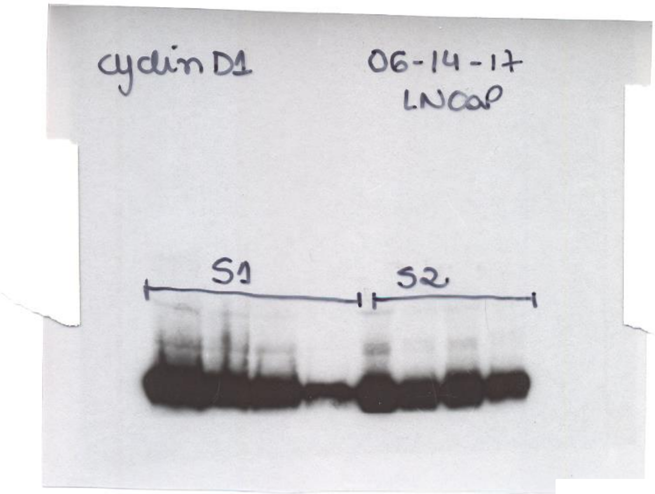

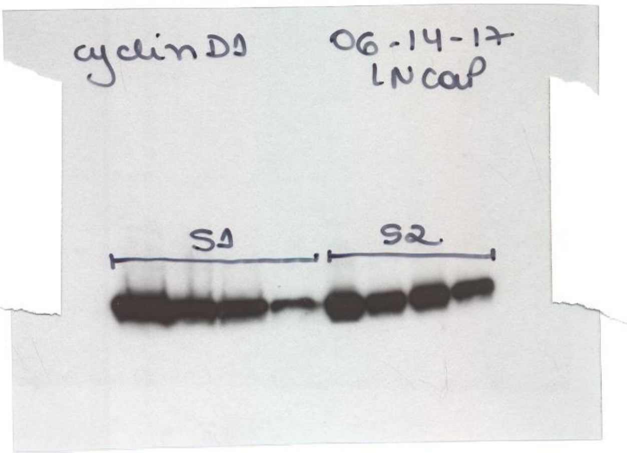

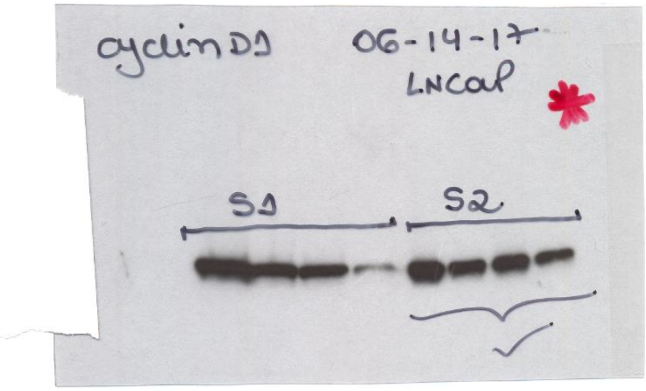

Supplementary information for Figure 2f **LNCaP** cells- **Cyclin D1** (31 KDa)
Blots marked with asterisk (red) and checked were used in main manuscript file

The sequence of samples in a lane is as- C, 5, 10, 25 µM NT
'S' represents different sample sets

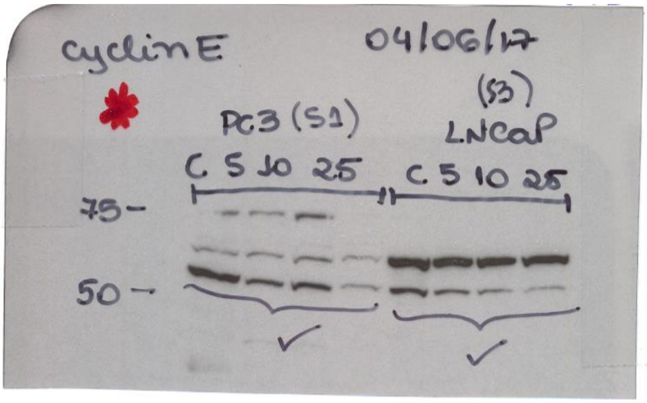

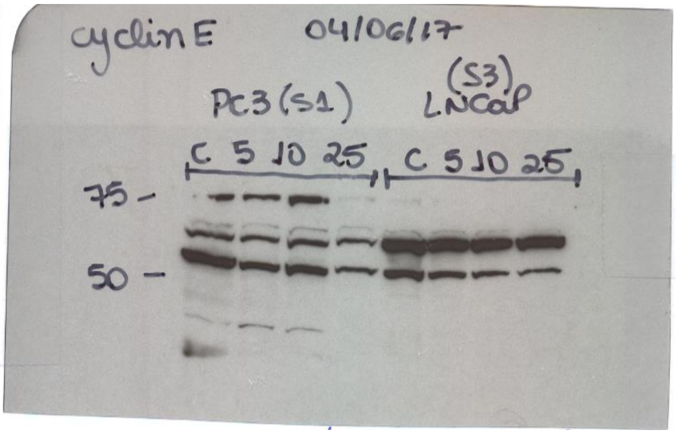

Supplementary information for Figure 2e **PC3** and Figure 2f **LNCaP** cells- **Cyclin E** (53 KDa). Blots marked with asterisk (red) and checked were used in main manuscript file

The sequence of samples in a lane is as- C, 5, 10, 25 µM NT
'S' represents different sample sets

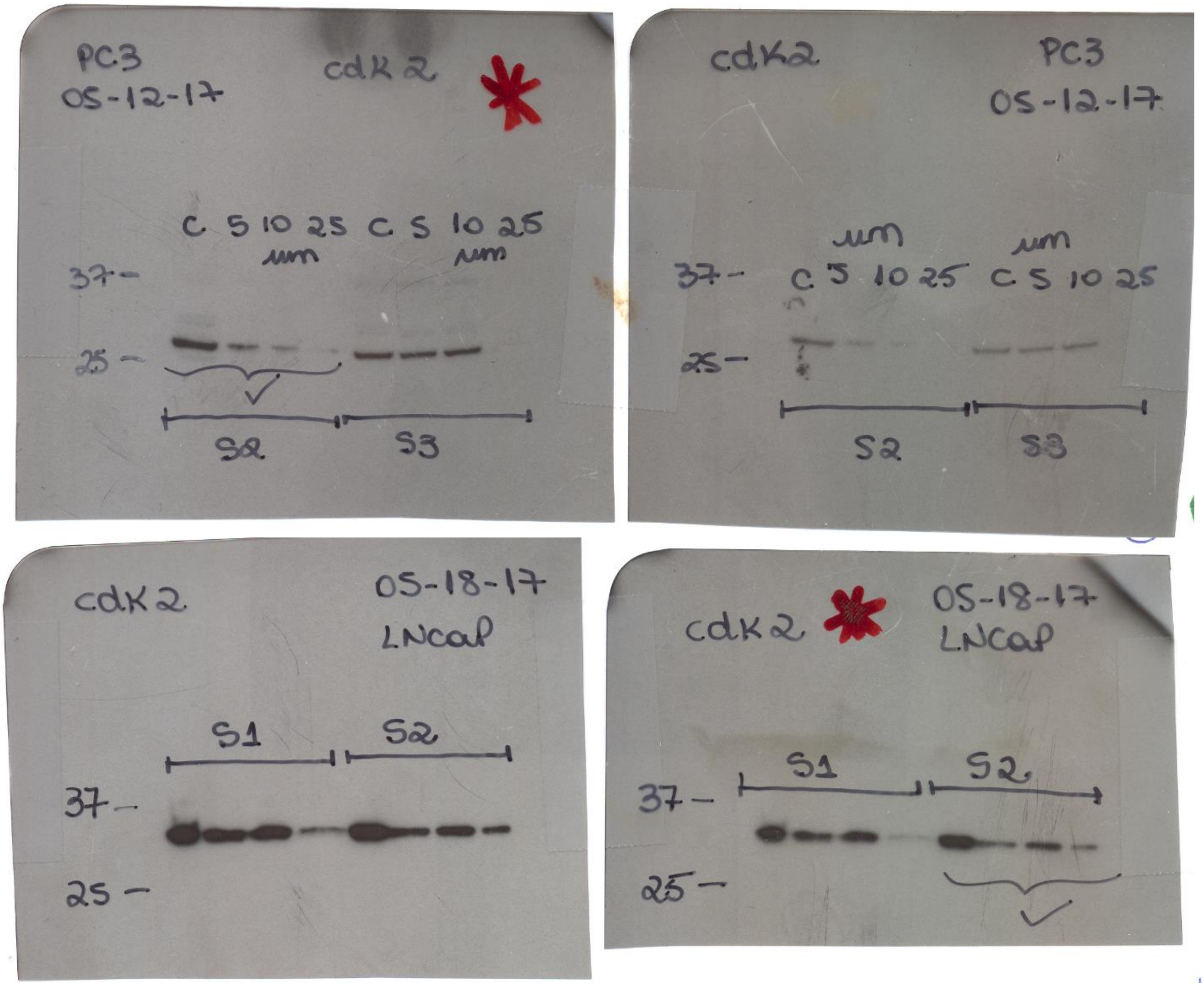

Supplementary information for Figure 2e **PC3** and Figure 2f **LNCaP** cells- **CDK2** (34 KDa). Blots marked with asterisk (red) and checked were used in main manuscript file

The sequence of samples in a lane is as- C, 5, 10, 25 µM NT
'S' represents different sample sets

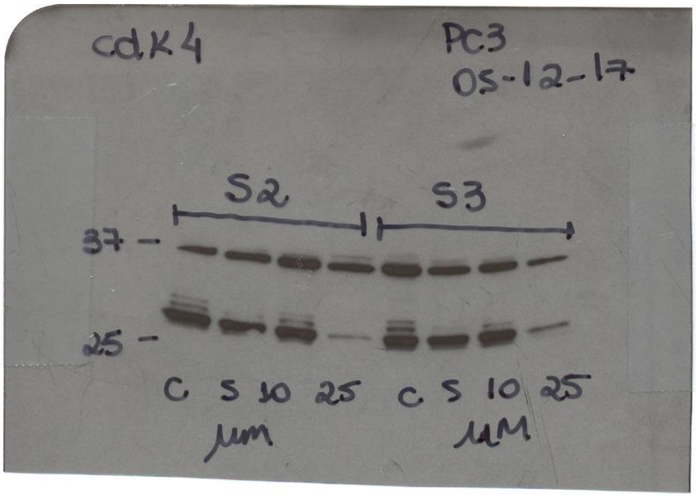

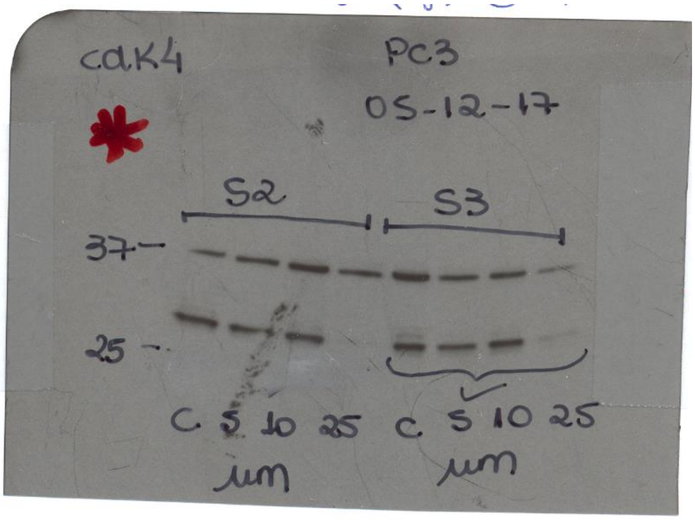

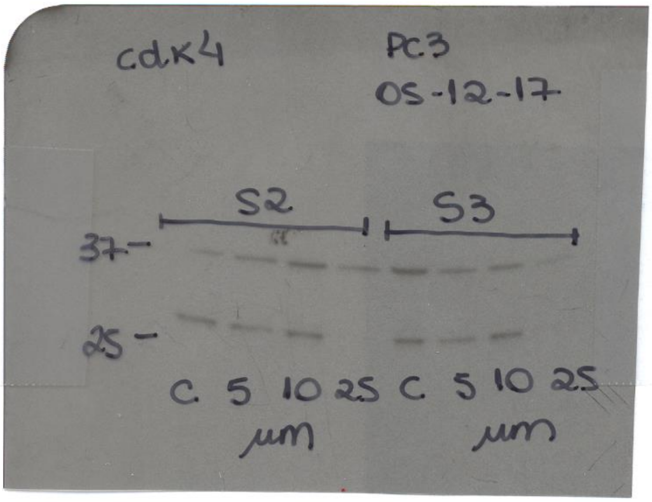

Supplementary information for Figure 2e **PC3** cells- **CDK4** (34 KDa)
Blots marked with asterisk (red) and checked were used in main manuscript file

The sequence of samples in a lane is as- C, 5, 10, 25 µM NT
'S' represents different sample sets

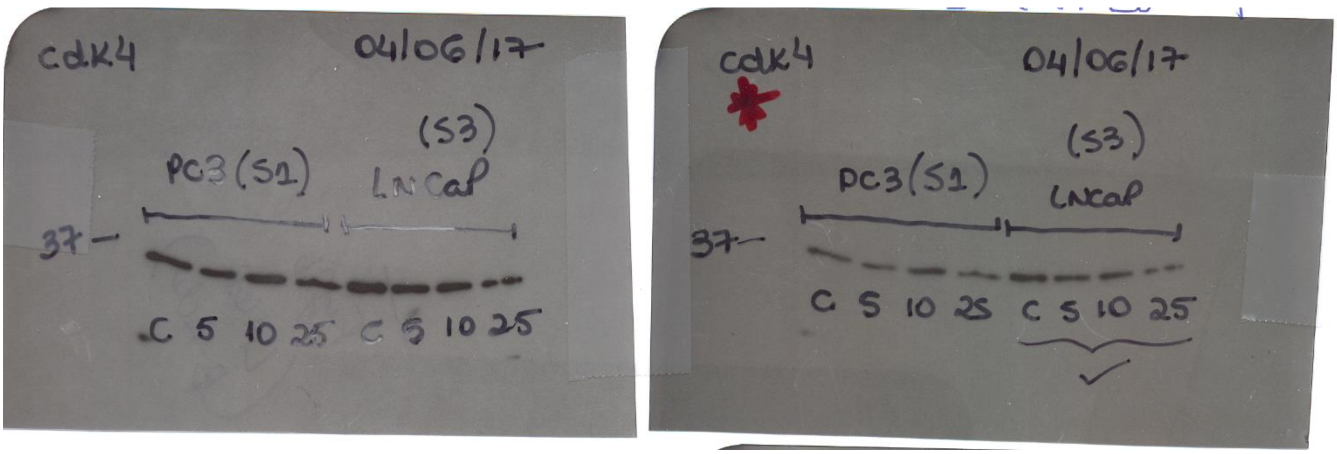

Supplementary information for Figure 2f **LNCaP** cells- **CDK4** (34 KDa)
Blots marked with asterisk (red) and checked were used in main manuscript file

The sequence of samples in a lane is as- C, 5, 10, 25 µM NT
'S' represents a sample set

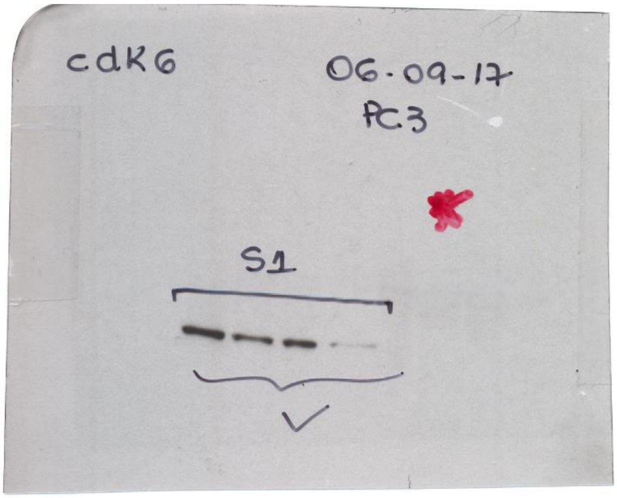

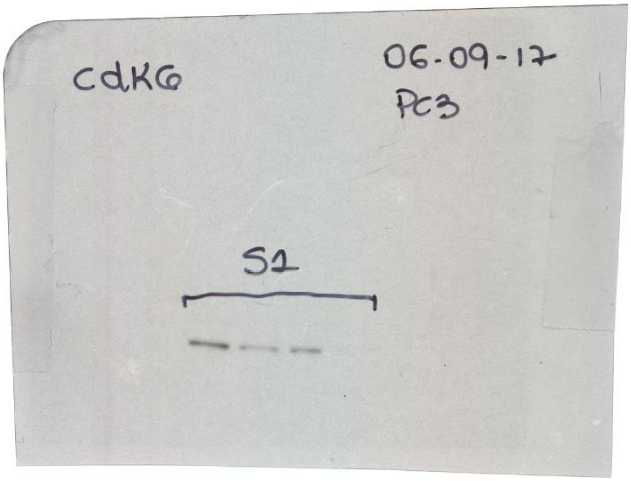

Supplementary information for Figure 2e **PC3** cells- **CDK6** (40 KDa)
Blots marked with asterisk (red) and checked were used in main manuscript file

The sequence of samples in a lane is as- C, 5, 10, 25 µM NT
‘S’ represents different sample sets

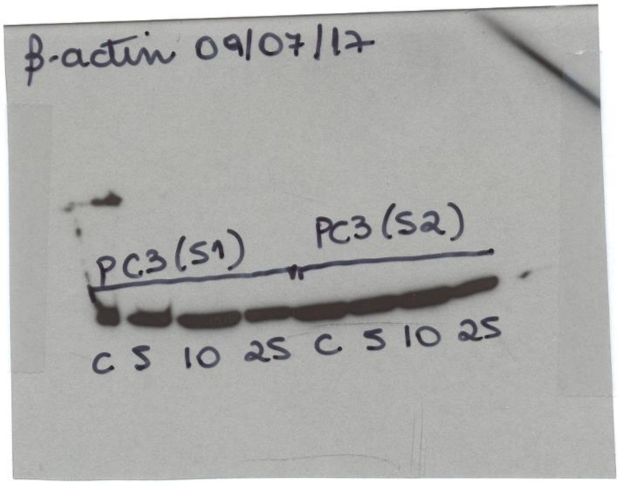

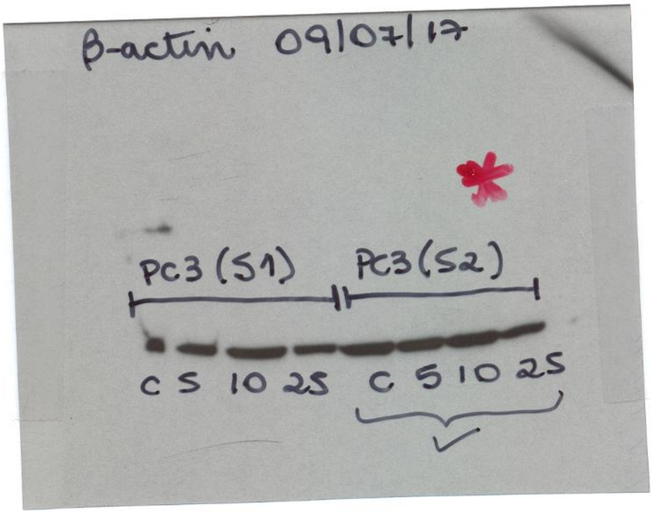

Supplementary information for Figure 2e **PC3** cells- **β Actin** (42 KDa)
Blots marked with asterisk (red) and checked were used in main manuscript file

The sequence of samples in a lane is as- C, 5, 10, 25 µM NT
'S' represents different sample sets

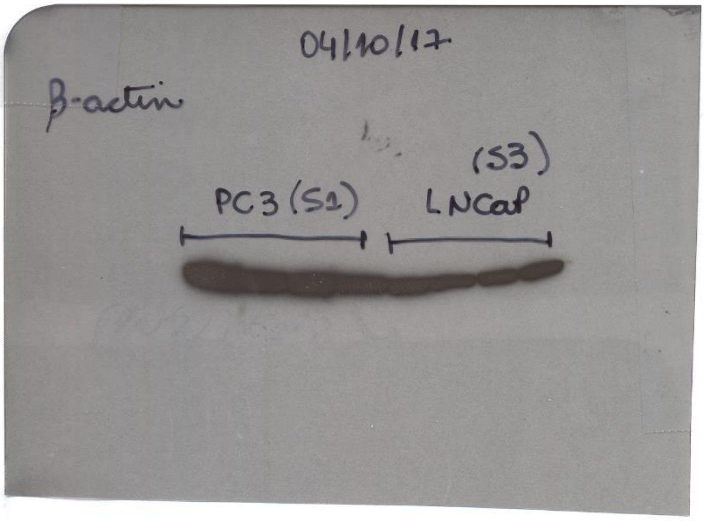

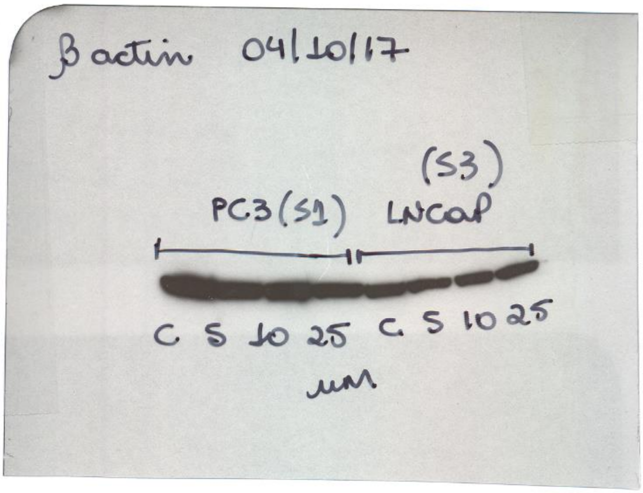

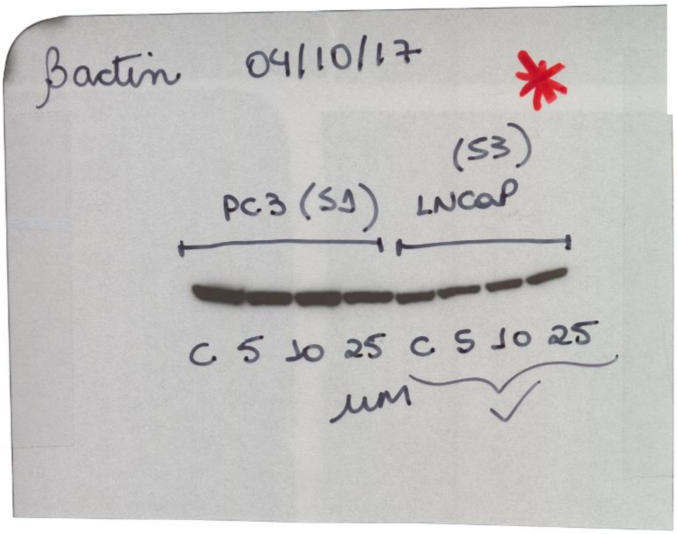

Supplementary information for Figure 2f **LNCaP** cells- **β Actin** (42 KDa)
Blots marked with asterisk (red) and checked were used in main manuscript file

Experiment 1- Top panel (7 min) exposure

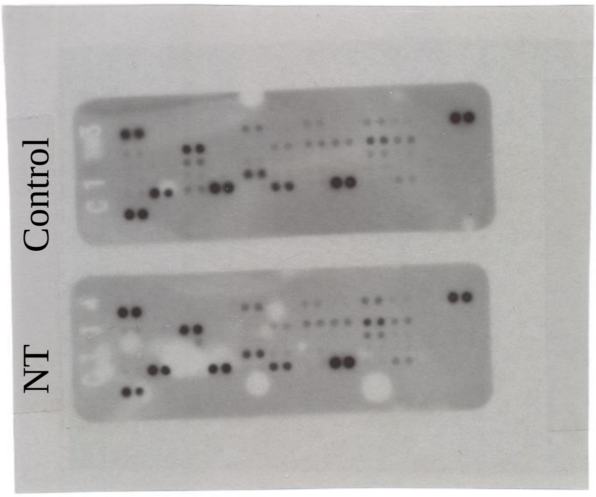

Experiment 1- Bottom panel (10 min) exposure

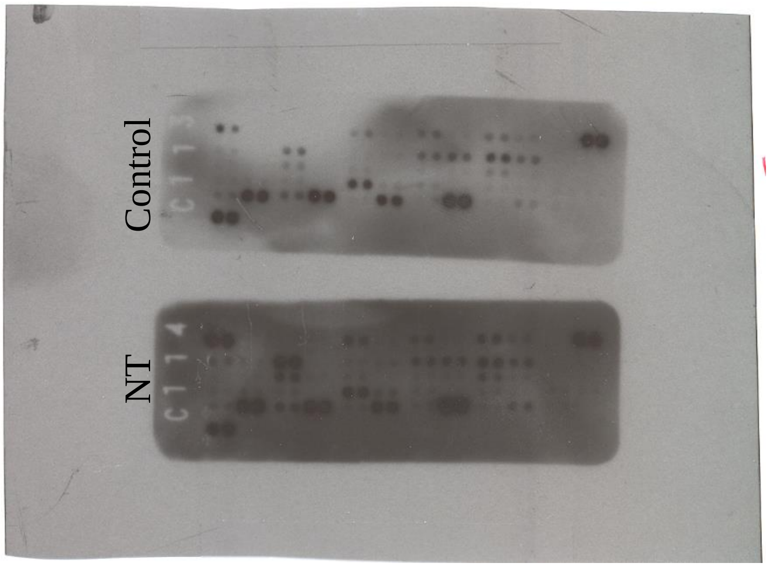

Supplementary information for **Figure 7a**

Experiment 2- Top panel (5 min) exposure

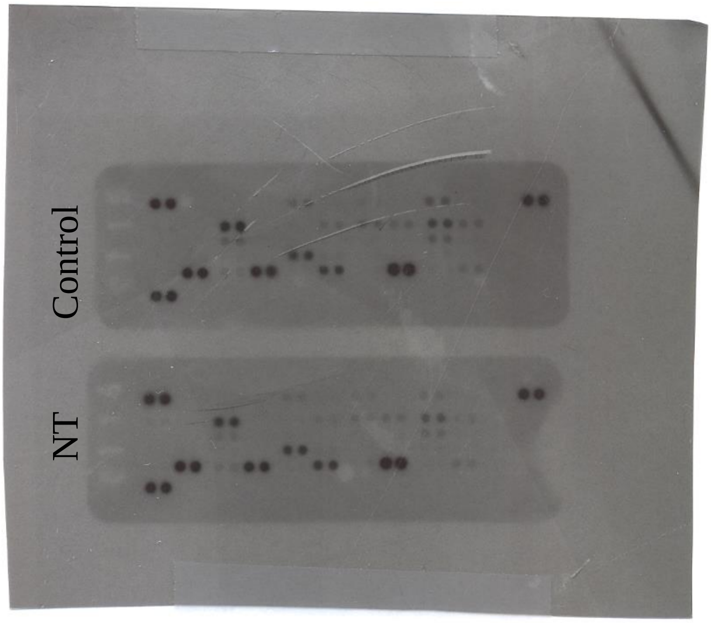

Experiment 2- Bottom panel (7 min) exposure

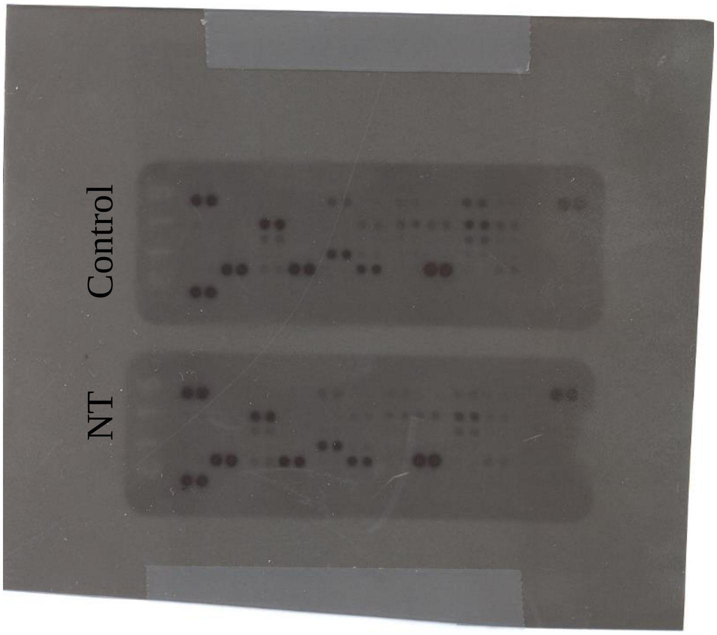

Supplementary information for **Figure 7b**

Antibodies used in the manuscript have been previously used by different research groups (Citation/publication details provided below)

Cyclin A (Santa Cruz - sc751):
RNF4 regulates DNA double-strand break repair in a cell cycle-dependent manner. Kuo, C-Y. *et al*. *Cell Cycle*. 2016;15(6):787-798. doi:10.1080/15384101.2016.1138184.

CDK2 (Santa Cruz - sc6248):
Impairment of primary cilia contributes to visceral adiposity of high fat diet-fed mice. Qiu, N. *et al*. *J Cell Biochem*. 2018;119: 1313–1325. https://doi.org/10.1002/jcb.26253.

CDK4 (Santa Cruz - sc749):
Amyloid β1-42 (Aβ1-42) Induces the CDK2-Mediated Phosphorylation of Tau through the Activation of the mTORC1 Signaling Pathway While Promoting Neuronal Cell Death. Lee, KH. *et al*. *Frontiers in Molecular Neuroscience*. 2017; 10:229. doi:10.3389/fnmol.2017.00229.

CDK6 (Santa Cruz - sc177):
Melatonin Suppresses the Growth of Ovarian Cancer Cell Lines (OVCAR-429 and PA-1) and Potentiates the Effect of G1 Arrest by Targeting CDKs. Srivastava SK, ed. Shen, C-J. *et al*. *International Journal of Molecular Sciences*. 2016;17(2):176. doi:10.3390/ijms17020176.

Cyclin E (Santa Cruz - sc198):
All-trans retinoic acid induces cell-cycle arrest in human cutaneous squamous carcinoma cells by inhibiting the mitogen-activated protein kinase-activated protein 1 pathway. Zhang, M.L. *et al.* 2014. *Clinical and experimental dermatology*. 39: 354-60; doi:10.1111/ced.12227.

Cyclin D1 (Santa Cruz (sc718) for Western Blotting and Thermo Scientific (#RM9104-S0) for IHC):
ß-catenin signaling is required for RAS-driven thyroid cancer through PI3K activation. Sastre-Perona A, *et al*. *Oncotarget*. 2016;7 (31):49435-49449. doi:10.18632/oncotarget.10356.
Consistent Immunostaining for Cyclin D1 can be achieved on a routine basis using a newly available rabbit monoclonal antibody. Cheuk, W. *et al*. *Am J Surg Pathol*. 2004; 28(6): 801-807. doi:10.1097/01.pas.0000126054.95798.94

E-Cadherin (Santa Cruz - sc21791):
hTERT peptide fragment GV1001 demonstrates radioprotective and antifibrotic effects through suppression of TGF-β signaling. Chen, W. *et al*. *International Journal of Molecular Medicine*. 2018;41(6):3211-3220. doi:10.3892/ijmm.2018.3566.

Vimentin (Santa Cruz - sc7557):
Lentiviral vector-mediated overexpression of mutant ataxin-7 recapitulates SCA7 pathology and promotes accumulation of the FUS/TLS and MBNL1 RNA-binding proteins. Alves, S. *et al*. *Molecular Neurodegeneration*. 2016;11:58. doi:10.1186/s13024-016-0123-2.

Ki67 (Abcam - ab15580):
Olfactory memory is enhanced in mice exposed to extremely low-frequency electromagnetic fields via Wnt/ß-catenin dependent modulation of subventricular zone neurogenesis. Mastrodonato, A. *et al. Scientific Reports*. 2018; 8:262.

Cleaved caspase 3 (Cell Signaling Technology #9661):
Adiponectin protects against lung ischemia-reperfusion injury in rats with type 2 diabetes mellitus. Li, D. *et al*. *Molecular Medicine Reports,* 2018(17), 7191-7201. https://doi.org/10.3892/mmr.2018.8748.

CD31 (Abcam - ab28364):
Exosomes from human umbilical cord blood accelerate cutaneous wound healing through miR-21-3p-mediated promotion of angiogenesis and fibroblast function. Hu, Y. *et al*. *Theranostics*. 2018;8(1):169-184. doi:10.7150/thno.21234.

Nestin (Santa Cruz - sc33677):
Neuronal Nitric Oxide Synthase in Neural Stem Cells Induces Neuronal Fate Commitment via the Inhibition of Histone Deacetylase 2. Jin, X. *et al*. *Frontiers in Cellular Neuroscience*. 2017;11:66. doi:10.3389/fncel.2017.00066.

VEGF (Santa Cruz - sc152):
Interleukin-17 promotes prostate cancer via MMP7-induced epithelial-to-mesenchymal transition. Zhang, Q. *et al*. *Oncogene*. 2017;36(5):687-699. doi:10.1038/onc.2016.240.
